# Tracking the Distribution and Burst of Nuclear Mitochondrial DNA Sequences (NUMTs) in Fig Wasp Genomes

**DOI:** 10.3390/insects11100680

**Published:** 2020-10-07

**Authors:** Jian-Xia Wang, Jing Liu, Yun-Heng Miao, Da-Wei Huang, Jin-Hua Xiao

**Affiliations:** 1 Institute of Entomology, College of Life Sciences, Nankai University, Tianjin 300071, China; jianxiamail@163.com (J.-X.W.); liujing_1987@yeah.net (J.L.); 2 Key Laboratory of Zoological Systematics and Evolution, Institute of Zoology, Chinese Academy of Sciences, Beijing 100101, China; fcbayernmyh@126.com

**Keywords:** NUMTs, mitochondrial DNA, transposable elements, evolution, fig wasps

## Abstract

**Simple Summary:**

Nuclear mitochondrial DNA sequences (NUMTs), which result from the insertion of exogenous mtDNA into the nuclear genome, are widely distributed in eukaryotes. However, how NUMTs are inserted into the nuclear genome and their post-insertion fates remain a mystery. Previous studies have suggested that Hymenoptera may be a group rich in NUMTs, which will be helpful to study the biological issues of NUMTs. We here select 11 species of fig wasps (Chalcidoidea, Hymenoptera) to analyze the distribution and evolution of NUMTs at the genomic level. The results show that the distributions of NUMTs are species- or lineage-specific. Furthermore, genomic environmental factors such as genome size, the damage-prone regions, and the mode of TE dynamics can determine the insertion and post-insertion fate of NUMTs. Especially because of TEs, the fragmentation and duplication of NUMTs, and thus their burst, are common. This is a relatively comprehensive investigation of the specific distribution of NUMTs and its influencing factors. Our study will help people to understand the evolution of exogenous fragments in the nuclear genome.

**Abstract:**

Mitochondrial DNA sequences can be transferred into the nuclear genome, giving rise to nuclear mitochondrial DNA sequences (NUMTs). NUMTs have been described in numerous eukaryotes. However, the studies on the distribution of NUMTs and its influencing factors are still inadequate and even controversial. Previous studies have suggested that Hymenoptera may be a group rich in NUMTs, in which we selected 11 species of fig wasps (Chalcidoidea, Hymenoptera) to analyze the distribution and evolution of NUMTs at the genomic level. The results showed that the contents of NUMTs varied greatly in these species, and bursts of NUMTs existed in some species or lineages. Further detailed analyses showed that the large number of NUMTs might be related to the large genomes; NUMTs tended to be inserted into unstable regions of the genomes; and the inserted NUMTs might also be affected by transposable elements (TEs) in the neighbors, leading to fragmentations and duplications, followed by bursts of NUMTs. In summary, our results suggest that a variety of genomic environmental factors can determine the insertion and post-insertion fate of NUMTs, resulting in their species- or lineage-specific distribution patterns, and that studying the evolution of NUMTs can provide good evidence and theoretical basis for exploring the dynamics of exogenous DNA entering into the nuclear genome.

## 1. Introduction

In eukaryotes, mitochondrial DNA sequences are frequently transferred into the nuclear genome, generating nuclear mitochondrial DNA sequences (NUMTs) [1]. NUMTs may mislead barcoding, phylogenetic and phylogeographic inferences when they are regarded as true mitochondrial sequences [2]. However, considering that the mutation rate of nuclear DNA is lower than that of the mitochondrial genome, NUMTs (as nuclear DNA) are often used as molecular fossils to infer and calibrate speciation events [3,4,5]. Most insect genomes have so far been reported to have very few or no copies of NUMTs. For example, no NUMTs have been detected so far in *Anopheles gambiae*, *Drosophila melanogaster* has only a few short NUMTs, and *Bombyx mori* has dozens of NUMTs [6,7,8,9]. However, in Hymenoptera, more than 1000 NUMTs have been found in honeybees [10]; 195 NUMTs have been found in *Nasonia vitripennis*, but, considering that the *Nasonia* mitochondrial genome is only partially reported, there should be more NUMTs [11]. Thus, Hymenoptera probably have more NUMTs than other insects.

It is generally believed that NUMTs are inserted into double-strand breaks (DSBs) in the nuclear genome via a nonhomologous end joining (NHEJ) mechanism [12,13,14]. Previous studies on the integration sites of NUMTs have found that although they are not randomly distributed in the genomes, and their insertion sites are different in different species. NUMTs in the human genome tend to be inserted into high-predicted DNA curvature and open chromatin regions, often adjacent to AT oligomers [15,16]; the integration of NUMTs in the human genome is concentrated in intron regions [17], which is similar to that seen in honeybee [18]. However, in the pig genome, most NUMTs are located mainly in intergenic regions and tend to be in regions of high GC content and near repetitive elements [19].

The number of NUMTs varies among different species [20,21], but the factors that result in variation in NUMT numbers among genomes are still unclear. The data on NUMTs insertion frequency from 85 eukaryotic genomes revealed a strong correlation between genome size and NUMT contents, which implies that the rate of NUMT insertion might be limited by the frequency of DSBs [22]. In addition, mutation or inactivation of gene *YME1* in yeast and humans can increase the escape rate of mtDNA, resulting in the burst of NUMTs [14,23,24]; however, NUMT bursts were not associated with relaxed purifying selection on *YME1* in birds [25]. Generally speaking, when the mtDNA is inserted into the nuclear genome as exogenous DNA, it will be eliminated in the process of natural selection. However, in some cases, duplication and fragmentation events will occur after the insertion of NUMTs into the genome [26,27], which is the key reason for the burst of NUMTs [28,29,30]. Misher et al. found that NUMTs that appear in several copies are duplicated along with flanking repeated elements [31]. Besides, many NUMTs are interrupted in some manner (i.e., they are not continuous with organelle DNA), and these insertions have been called a “complex”, which makes no statement on the issue of whether the discontinuity arose during the process of insertion or through subsequent recombinational events. In principle, the fragmentation events can result from DNA insertions into the NUMT, and the insertions separating the mtDNA-derived sequences should, in the simplest case, be some kind of mobile DNA [30]. Based on the analysis results of NUMTs in six plant genomes, Michalovova et al. proposed a model of how transposable elements (TEs) can be involved in the evolution of NUMTs: after new mtDNA sequences are integrated into the genome, the insertion of TEs can make them fragmented, and TE-based recombination can result in deletion, replication, or movement of these NUMT fragments [32]. However, whether the hypothesis can be applied to more taxa remains to be verified.

In fact, the generation of NUMTs includes the processes of the escape of mtDNA, the integration into the nuclear genome, and the dynamics of post-insertion [22], each of which will be affected by different or even multiple factors and then produce different results. At present, the research on species-specific distribution of NUMTs and its influencing factors is still at the exploratory stage. Most of the relevant reports only focus on a part of the process of NUMT production, or one or two influencing factors, and some results need to be verified by adding more taxa. Species with sufficient NUMT contents will help us to explore the distribution and dynamic evolution of NUMTs. In Hymenoptera, NUMTs may be abundant, but they have been extensively studied in only two species: *Apis mellifera* and *N. vitripennis*. In our lab, by a combination of second- and third-generation sequencing methods, we have obtained high-quality genome assembly data of 11 species of fig wasps (Chalcidoidea, Hymenoptera), which provide us with a good opportunity to study the NUMTs of Hymenoptera. In the present study, based on the genome data of the 11 fig wasps, we not only reveal the species- and lineage-specific patterns of the distribution and burst of NUMTs but also, benefiting from the sufficient amount of data, comprehensively decipher the origins of these specific distribution patterns. This will help us to understand the evolution of exogenous fragments in the nuclear genomes.

## 2. Materials and Methods

The whole nuclear genome and mitochondrial genome sequences of the following 11 fig wasps were obtained in our lab: *Dolichoris vasculosae*, *Wiebesia pumilae*, *Eupristina koningsbergeri*, *Platyscapa corneri*, *Ceratosolen fusciceps*, *Kradibia gibbosae*, *Sycobia* sp.2, *Apocrypta bakeri*, *Philotrypesis tridentata*, *Sycophaga agreansis*, *Sycophila* sp.2. The whole bodies of fig wasps were used for DNA extraction for each species. Genomic DNA were extracted by LiAC precipitation method [33]. Two libraries with insert sizes of 450 bp and 20 kb were constructed, and then sequencing was performed on Illumina Hiseq2500 (Illumina Technologies, San Diego, CA, USA) and Pacific Biosciences (Pacific Biosciences, Menlo Park, CA, USA) platforms. The Illumina sequencing and PacBio sequencing data were assembled using SMRTdenovo (v1.0) (https://github.com/ruanjue/smartdenovo). Gene functions were assigned according to the best match by aligning protein sequences predicted to KEGG (https://www.genome.jp/kegg/), NR (https://www.ncbi.nlm.nih.gov), Swiss-Prot (https://www.uniprot.org), eggNOG (http://eggnog5.embl.de), and GO (http://geneontology.org) databases. Gene functions were predicted from the best blastp (e-value < 10^−5^) hits in SwissProt database. The Illumina sequencing data were assembled by CAP3 software [34] to obtain mitochondrial genome. and genes were annotated using MITOS web server [35] (unpublished data [36]). Accession numbers for the mitochondrial genomes are as follows: MT947596, MT947601, MT947597, MT947604, MT916179, MT947598, MT947600, MT906648, MT947602, MT947599, and MT947603. Accession numbers for the nuclear genomes are as follows: JACCHY000000000, JACCHZ000000000, JACCHV000000000, JACCHW000000000, RCIC00000000, JACCHX000000000, JACCIC000000000, JACCIA000000000, JACCIB000000000, JACCID000000000, and JACCIE000000000.

**Figure 1:**
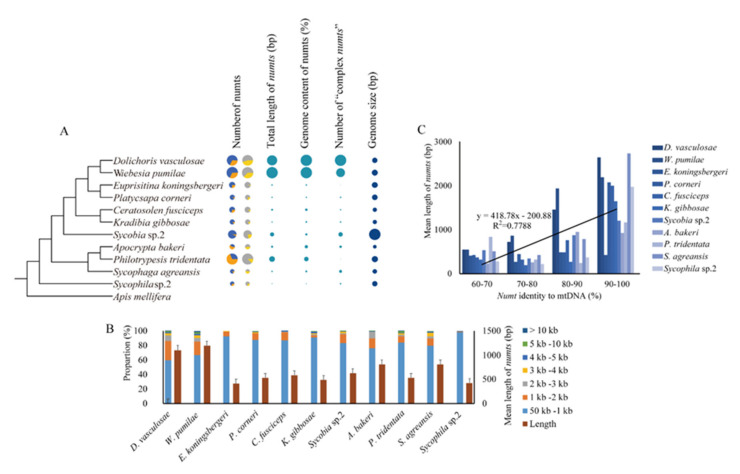
Distribution of nuclear mitochondrial DNA sequences (NUMTs) in fig wasp genomes. (**A**) The number of NUMTs in the fig wasps. The phylogenetic tree on the left was obtained from our unpublished phylogenomic work [36], and the pie chart columns on the right display the statistical results of the NUMTs. The size of the pie chart corresponds to the number of data in each group. In the result group of “Number of NUMTs”, the pie chart in the first column shows the content of “duplicate type” NUMTs (orange) and “insertion type” NUMTs (blue), while the second column shows the content of NUMTs contained in “complex NUMTs” (yellow) compared to others (grey). (**B**) The length distribution of NUMTs. Each species had two columns, with the left one representing the proportion of NUMTs in different length ranges (corresponding to values on the left vertical axis) and the right one representing the mean length of NUMTs in the species (corresponding to the values on the right vertical axis). (**C**) The correlation between NUMT length and the sequence divergence of NUMTs from their corresponding mtDNAs. For each species, the average lengths of NUMTs of different ranges of similarity with their mtDNA are shown, and in general, the higher the similarity to the corresponding mtDNA, the longer the average NUMT length.

To identify NUMTs in each species, mitochondrial genome was used as query for blastn against nuclear genome. The search was done using blast v2.10.0+ with e-value < 10^−4^, while removing the hits of less than 50 bp. We chose the threshold of 10^−4^, as it was suggested in Tsuji et al.’s study that this is a reasonable e-value [16]. We mapped the length, number, and the percentage of NUMTs onto an 11-species fig wasp tree (Figure 1A), which was extracted from our unpublished phylogenomic work. The NUMT annotations are provided as Supplementary Material. The NUMT data of other insect species, including the 4 other Hymenoptera species (*Pteromalus puparum*, *Apis cerana*, *A. mellifera*, and *N. vitripennis*) and 20 species from other orders of Diptera (11 species of the *Drosophila* genus, *A. gambiae*, and *Aedes aegypti*), Lepidoptera (*Melitaea cinxia*, *Manduca sexta*, *Heliconius melpomene*, *Danaus plexippus*, *Chilo suppressalis*, and *Bombyx mori*), and Coleoptera (*Tribolium castaneum*), were obtained from previous works [8,9,11,22,34].

The genomic context of the detected NUMTs was analyzed, assessing the following factors: (1) the AT contents of 250 bp upstream and downstream flanks of each NUMT and 50 bp at both ends of NUMT Bedtools software (available from: https://bedtools.readthedocs.io/en/latest/) was used to calculate mean AT content with 5 bp sliding window), (2) the position of insertion (intergenic, intronic, and coding regions), and (3) the density and number of TEs. We chose all scaffolds containing NUMTs in the genome to calculate NUMT and TE density with a 50 kb sliding window by using Perl script (Perl script was written by colleagues in our lab). The number of TEs in the vicinity of NUMTs (5 kb) was estimated using the RepeatMasker-4.1.0 software (available from: http://www.repeatmasker.org/) to investigate the features of the integrated regions of NUMTs. In all, 5 kb sequences from both flanking regions of NUMTs were extracted. The number of each type of TE within the regions was estimated using RepeatMasker with parameters of ‘-lib -s -nolow -norna -engine ncbi -no is’. The ‘-lib’ is an annotated database of repetitive sequences of each fig wasp, and data were obtained by using RepeatModeler and RepeatMasker (unpublished data [36]).

Pairs of NUMTs and their flanking DNA were compared to identify the “duplicate NUMTs” arisen from DNA duplication. If one NUMT arose from another by DNA duplication in the nucleus, homology between the NUMTs should extend into the nuclear DNA that flanks them, and the degree of similarity between the NUMTs and between flanking DNA regions should be the same. The length and percentage similarity of NUMTs and flanking DNA homology (if present) were determined using blastn.

In the identification of “complex NUMTs”, all hits identified by blastn that fulfilled the following three criteria were considered to be part of one insertion event and thus included in the list of “complex NUMTs”: (1) NUMTs separated by < 10 kb of DNA of nonmitochondrial origin were considered as a cluster; (2) the NUMTs in the same cluster had a very good synteny relationship with the corresponding mitochondrial region; (3) the direction of the NUMT sequence within the same cluster was consistent with the corresponding mitochondrial region.

We used phylogenetic generalized least squares (PGLS) regression [37] to investigate the relationship between the length, number, and the percentage of NUMTs and genome size while statistically controlling for phylogeny. PGLS regression analyses were performed using R with the caper packages (available online at: http://CRANRproject.org/package=caper).

Genes with loss-of-function alleles that lead to an increased rate of “mitochondrial DNA escape” are known from the budding yeast *Saccharomyces cerevisiae*, and one of these loci is *YME1*. To statistically test whether *YME1* genes were under relaxed purifying selection in four species (*W. pumilae*, *P. tridentata*, *D. vasculosae*, and *Sycobia* sp.2) with NUMT burst, we used the likelihood ratio test in RELAX [38], comparing the model fixing k = 1 and the model allowing k to be estimated. The four species (*W. pumilae*, *P. tridentata*, *D. vasculosae*, and *Sycobia* sp.2) branches were alternatively set as the foreground branch for the test.

## 3. Results

### 3.1. Identification of NUMTs in the Fig Wasp Genomes

We searched for NUMTs in 11 fig wasp genomes by using blastn, and the results showed that the greatest number of NUMTs was present in *W. pumilae* (628 NUMTs), followed by *P. tridentata* (618 NUMTs), *D. vasculosae* (590 NUMTs), and *Sycobia* sp.2 (426 NUMTs), and the least was present in *S. agreansis* (109 NUMTs). The longest total length of MUNTs was present in *W. pumilae* (752,110 bp), followed by *D. vasculosae* (671,621 bp), *P. tridentata* (374,545 bp), and *Sycobia* sp.2 (265,482 bp), and the shortest was present in *Sycophila* sp.2 (246,620 bp). In terms of the percentage of NUMTs in the genome, the highest genome content of NUMTs was 0.235% in both *W. pumilae* and *D. vasculosae*, followed by *P. tridentata* (0.094%) and *A. bakeri* (0.065%), and the lowest was in *Sycophila* sp.2 (0.016%) (Figure 1A, Table S1). Therefore, the number and length of NUMTs in *W. pumilae*, *P. tridentata*, *D. vasculosae*, and *Sycobia* sp.2 all showed a burst trend. Among them, *W. pumilae* and *D. vasculosae* were in the same evolutionary lineage, and they had the longest length and highest genome content of NUMTs. Therefore, it can be seen that the number and length of NUMTs varied remarkably across different species, showing distribution of not only species-specificity but also lineage-specificity. We further carried out correlation analysis between the length, number, and the percentage of NUMTs in the genome and genome size for the 11 fig wasps. When the phylogenetic background is not considered, the length, number, and the percentage of NUMTs were not correlated with genome size, with no significant correlation (*p* = 0.525, 0.154, and 0.982). Considering the phylogenetic background, PGLS analysis showed that there were also no significant correlations between the length (*R*^2^ = 0.0187, *p* = 0.6884), number (*R*^2^ = 0.3505, *p* = 0.055), and the percentage (*R*^2^ = 0.0988, *p* = 0.3466) of NUMTs and genome size. However, *Sycobia* sp.2, with the largest genome, and *P. tridentata*, with the second largest genome, were indeed rich in NUMT number and length.

In the process of NUMT generation, some of them arise directly from the insertion of mtDNA into the nuclear genome (we named them “insertion type”), while some NUMTs are produced by duplication of NUMTs that have been inserted into the nuclear genome (we named them “duplicate type”). For these “duplicate type” NUMTs, some nuclear DNA sequences on its both flanks are often included in their repetitive duplications and insertions; therefore, sequence similarity will extend to the flanking nuclear DNA sequences when sequence alignments are demonstrated [39], and based on this, we can identify those “duplicate type” NUMTs (Figure 1A, Table S1). Among the studied 11 fig wasp species, the largest number of “duplicate type” NUMTs was present in *P. tridentata* (425 NUMTs), followed by *W. pumilae* (202 NUMTs) and *D. vasculosae* (193 NUMTs), and *P. corneri* (15 NUMTs) had the least. The proportion of “duplicate type” NUMTs in all NUMTs varied among the species, with the largest proportion present in *P. tridentata* (68.77%) and *A. bakeri* (60.87%), followed by *D. vasculosae* (32.71%) and *W. pumilae* (32.17%), and the least in *Sycobia* sp.2 (7.04%). It can be seen that the distribution of “duplicate type” NUMTs showed lineage-specificity: *P. tridentata* and *A. bakeri* had the highest proportion, and *D. vasculosae* and *W. pumilae* were the second in number and proportion (Figure 1A, Table S1). For each species, we could get the number of “insertion type” NUMTs after removing the “duplicate type” NUMTs from the total NUMTs. As for the comparison of the number of “insertion type” NUMTs, *W. pumilae* (426 NUMTs) had the largest number of “insertion type” NUMTs, followed by *D. vasculosae* (397 NUMTs), *Sycobia* sp.2 (396 NUMTs), and *P. tridentata* (193 NUMTs), and *A. bakeri* had the smallest (63 NUMTs). Therefore, species with more “insertion type” NUMTs also showed lineage-specific characteristics (Figure 1A, Table S1).

We investigated the length distribution of NUMTs and their similarity to mitochondrial genes, and the results showed that in all species, most identified NUMTs were short fragments (mean lengths ranged from 299 bp for *Sycophila* sp.2 to 1231 bp for *W. pumilae*), and many of them were shorter than 1000 bp (58.5 % in *D. vasculosae* to 98.2 % in *Sycophila* sp.2) (Figure 1B). However, in some species, the longest NUMTs were more than 10 kb in length, such as in *D. vasculosae* (12,107 bp), *W. pumilae* (12,250 bp), *P. tridentata* (14,100 bp), and *Sycophila* sp.2 (14,898 bp). We further compared NUMT length and the sequence divergence of NUMTs from their corresponding mtDNAs, and the Spearman correlation coefficient was 0.88, with a strong significant correlation (Wilcoxon test, *p* = 1.58 × 10^-5^). The results showed that longer NUMTs tended to be more similar to their corresponding mtDNA sequences (Figure 1C), implying their recent origin.

**Figure 2:**
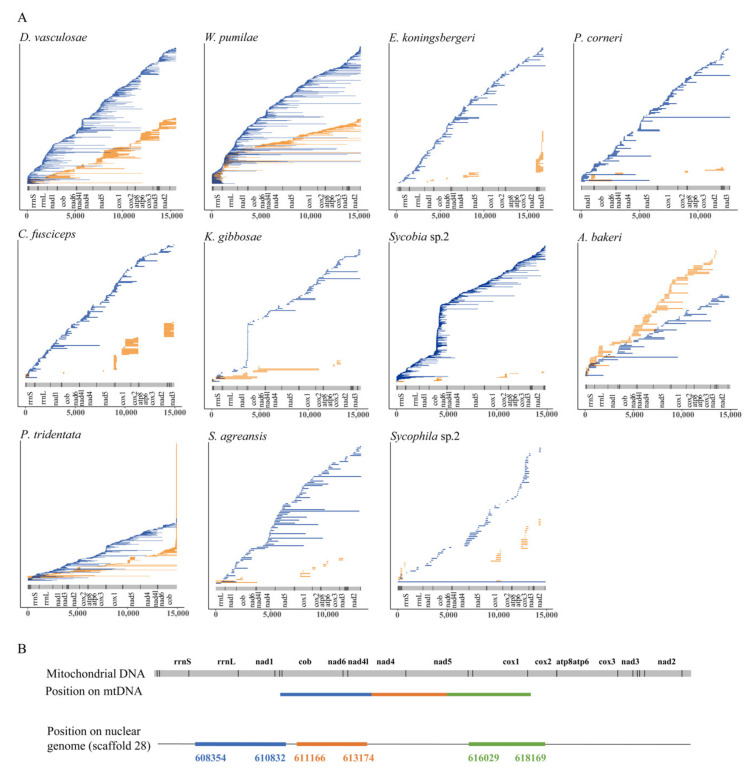
Mapping of NUMTs and their mitochondrial genome regions (**A**) and a detailed display of one example of fragmentation event in the species of *A. bakeri* (**B**). (**A**) For each species, the NUMTs were mapped onto their original positions on the mitochondrial genome (the length and mitochondrial genes are shown in the bottom). The orange lines represent the “duplicate type” NUMTs and the blue lines represent “insertion type” NUMTs. (**B**) The picture shows a 9815 bp long sequence containing three NUMTs (in different colors) on scaffold 28 in the genome of *A. bakeri* (in the bottom) and the origin of the three NUMTs on its mitochondrial genome (on the top). The numbers show the start and end positions of different NUMTs on the scaffold. On the mitochondrial genome, transfer RNA genes are indicated with vertical lines. Acronyms: nad1-6, NADH dehydrogenase subunits 1 to 6; cox1–cox3, cytochrome c oxidase subunits I to III; atp6 and atp8, ATP synthase 6 and 8; cob, cytochrome b; rrnL and rrnS, 16S and 12S rRNA.

For each species, when we mapped all the NUMTs to their corresponding mitochondrial genome, we found that NUMTs might originate from any region of the mitochondrial genome (Figure 2A). In addition, we also noticed that some adjacent NUMTs, which we called “complex NUMTs”, might be derived from the fragmentation of the same “insertion type” NUMT (e.g., in Figure 2B) [30], so we used strict criteria to identify and count the number of “complex NUMTs” in each species. As a result, 196 “complex NUMTs” were identified in 11 fig wasps, with the numbers varying among different species: the largest number (55) was found in *D. vasculosae*, and the smallest (2) was found in *E. koningsbergeri*. In particular, in the lineage of *D. vasculosae* and *W. pumilae*, both of them had the largest number of NUMTs and the largest number of “complex NUMTs”, indicating that fragmentation had contributed many NUMTs to their genomes (Figure 1A, Table S1).

### 3.2. Characteristics of NUMT Insertion Regions in the Genomes

**Figure 3:**
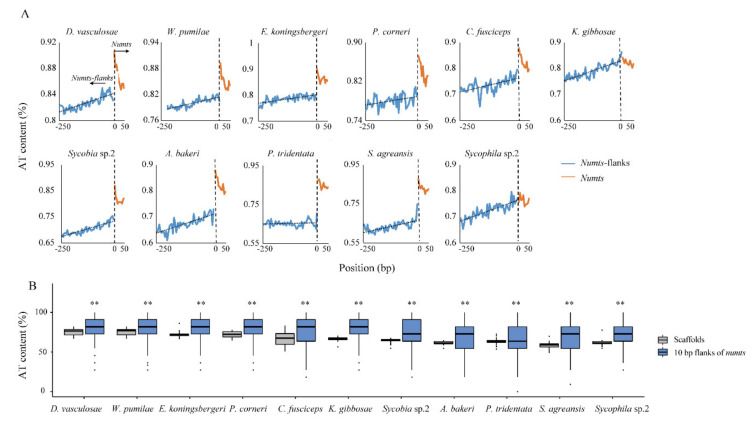
Mean AT content in NUMT flanking sequences. (**A**) For each species, the mean AT content (5 bp windows) of 250 bp flanks of all NUMTs (blue lines) and 50 bp at both ends of all NUMTs (orange lines) are displayed. The horizontal axis gives the positions of NUMTs and NUMT flanking regions in genome. The vertical axis gives the mean AT content at each position. (**B**) Comparison of mean AT content in the 10 bp flanking regions of all NUMTs to the mean AT content of all scaffolds containing NUMTs in the genome. ** *p* < 0.01, Wilcoxon test.

In order to explain the specific distribution of NUMTs and its influencing factors, we analyzed the genomic environment where NUMTs were inserted. First, the AT content of NUMT flanking sequences was analyzed. The results showed that in all species, the closer to NUMTs, the higher the AT content was (Figure 3A). In each species, the 10 bp flanking regions of the NUMT sequences showed AT content that was significantly higher than the average AT content in the scaffold in which they are located (Wilcoxon test, *p* < 0.01) (Figure 3B).

When we surveyed the position information of NUMTs in the nuclear genome, we found that more than half of the NUMTs of each fig wasp species were located in intergenic regions. Among them, the proportion was as high as 94.2% in *P. tridentata*, while it was only 51.4% in *S. agreansis*, which was the lowest. Except for the NUMTs in intergenic regions, the rest of the NUMTs in each species were located in the intronic regions within genes. In the *D. vasculosae* genome, a total of 147 out of the detected 490 NUMTs were located within 69 different annotated genes, which was the largest number of NUMTs located in the intronic regions in these species. The least was found in *P. tridentata* (36 NUMTs). No NUMTs were found in the protein-coding regions in all of the species (Table 1).

**Figure 4:**
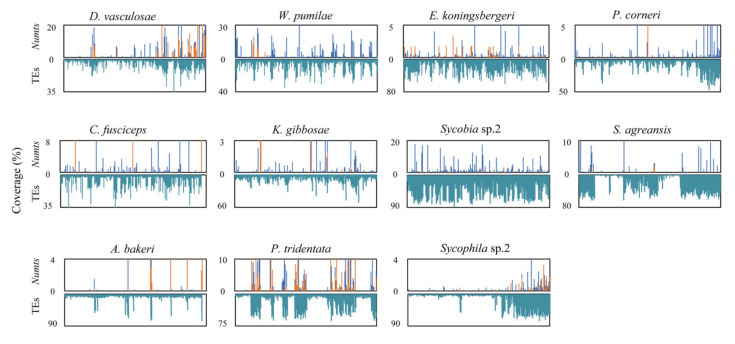
The density distribution of NUMTs and transposable elements (TEs) on the genome. The results of each species are shown in separate panels, with the distribution of “insertion type” (as labeled in blue color) and “duplicate type” (as labeled in orange color) NUMTs on top of the panel and that of total TEs (including DNA transposons, long interspersed nuclear elements (LINEs), short interspersed nuclear elements (SINEs), rolling cycles (RCs), and long terminal repeats (LTRs)) at bottom. The sliding window size was set to 50 kb. The horizontal axis represents position of the windows in the nuclear genome, and the vertical axis represents the coverage of NUMTs or TEs in the sliding windows.

We also studied the correlation between the distributions of NUMTs and TEs. The distribution densities of TEs and NUMTs were surveyed based on 50 kb windows, with TEs including DNA transposons, long interspersed nuclear elements (LINEs), short interspersed nuclear elements (SINEs), rolling cycles (RCs), and long terminal repeats (LTRs). As shown in Figure 4 in most species, the pattern of NUMT distribution was similar to that of TEs; i.e., where the density of NUMTs was high, the density of TEs was also high (Figure 4).

### 3.3. Relationship between Duplication/Fragmentation Events of NUMTs and TEs

**Figure 5:**
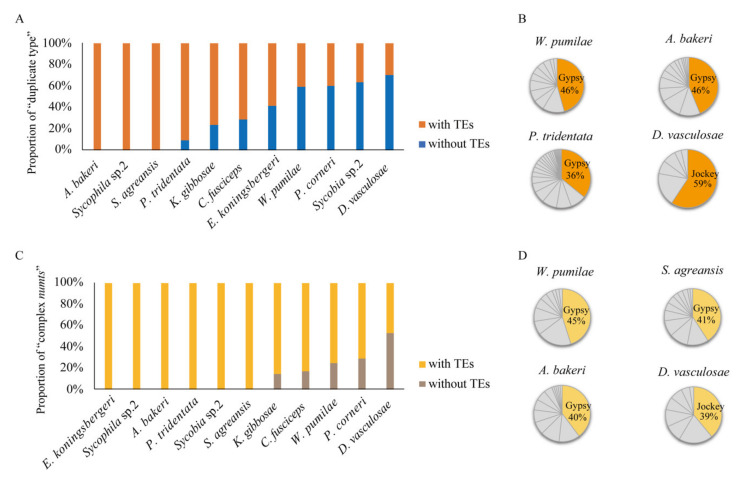
Correlation of TEs with “duplicate type” NUMTs and “complex NUMTs”. (**A**) The proportion of “duplicate type” NUMTs with or without TEs on the flanking sequences. (**B**) Pie charts showing the content of different TE superfamilies in flanking regions of “duplicate type” NUMTs in the four species *W. pumilae*, *A. bakeri*, *P. tridentata*, and *D. vasculosae*. The colored areas represent the TE type with the highest proportion in each species. (**C**) The proportion of “complex NUMTs” with or without TEs on the interior and flanking sequences. (**D**) Pie charts showing the content of different TE superfamilies in flanking or interior regions of “complex NUMTs” in the four species *W. pumilae*, *S. agreansis*, *A. bakeri*, and *D. vasculosae*. The colored areas represent the TE type with the highest proportion in each species. In (**B**) and (**D**), the results of TE type proportions of only four species are shown, because the number of TEs of the other species is small, or the proportions of different TEs types are very similar.

As mentioned above, there were “duplicate type” NUMTs and “complex NUMTs” in all fig wasp species. Coupled with the similar density distribution of NUMTs and TEs in genomes, we further studied the distribution of TEs near “duplicate type” NUMTs and “complex NUMTs” in order to obtain more evidence about the correlation between NUMTs and TEs. By using RepeatMasker software, we analyzed whether there were TEs on the flanks (<5 kb) of “duplicate type” NUMTs. As a result, in the three species of *A. bakeri*, *S. agreansis*, and *Sycophila* sp.2, TEs existed on the flanks of all “duplicate type” NUMTs (Figure 5A), while in the other species, the proportion of “duplicate type” NUMTs containing TEs on the flanking sequences varied, and the lowest was found in *D. vasculosae* (30%). Furthermore, we counted the TE types closest to “duplicate type” NUMTs. The result showed that the largest proportions of TEs were Gypsy in *W. pumilae*, *A. bakeri* and *P. tridentata*, accounting for 46%, 44%, and 36%, respectively. In *D. vasculosae*, Jockey had the largest proportion (59%) of TEs closest to “duplicate type” NUMTs (Figure 5B).

As mentioned above, *P. tridentata* and *A. bakeri*, the two species of Pteromalidae, had the highest proportion of “duplicate type” NUMTs in all species, but the number of “duplicate type” NUMTs in *A. bakeri* (98 NUMTs) was much less than that in *P. tridentata* (425 NUMTs) (Figure 1A, Table S1). Further analysis showed that the NUMT burst in *P. tridentata* was mainly caused by a large number of duplications of NUMTs corresponding to the 14,885–14,950 bp region of its mitochondrial genome (Figure 2A). In terms of the TEs in the flanking sequences of “duplicate type” NUMTs, more than 90% of the NUMT flanks contained TEs in both species (Figure 5A), and the number of LTR/Gypsy was the largest (Figure 5B). Considering this in combination with the fact that the Gypsy sequences accounted for 6.866% and 1.290% of the total genomes of *P. tridentata* and *A. bakeri*, respectively (data from our unpublished phylogenomic work), it was speculated that the burst of “duplicate type” NUMTs in *P. tridentata* was closely related to the abundance of Gypsy in its genome.

When we searched for TEs within and around “complex NUMTs”, we found that there were six species (*A. bakeri*, *E. koningsbergeri*, *P. tridentata*, *Sycobia* sp.2, *Sycophila* sp.2, and *S. agreansis*) in which all of the “complex NUMTs” had TEs inside or around, and, in the remaining species, more than 70% of the “complex NUMTs” had TEs inside or around, except for *D. vasculosae*, with only 47% (Figure 5C). When we surveyed the type of these TEs, the results showed that the species with the largest proportions of Gypsy were *W. pumilae* (45%), *S. agreansis* (41%), and *A. bakeri* (40%), while *D. vasculosae* had the largest proportion of Jockey (39%) (Figure 5D).

## 4. Discussion

**Figure 6:**
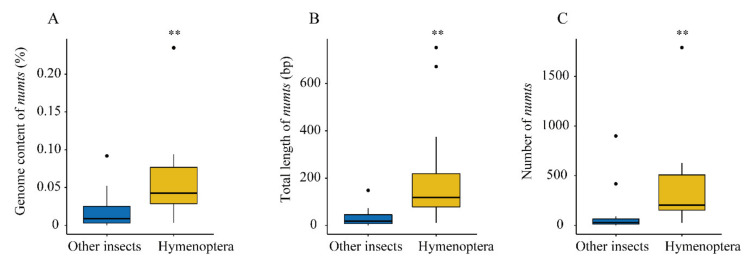
Comparison of NUMTs in Hymenoptera and other insects (Diptera, Lepidoptera, and Coleoptera). The content of NUMTs in the genomes (**A**), the total length of NUMTs (**B**), and the number of NUMTs (**C**) are compared. ** *p* < 0.01, Wilcoxon test.

In this study, 11 fig wasp species were selected to analyze the number and distribution of NUMTs in their genomes. This is the first detailed and systematic comparative study of the number and distribution of NUMTs at the genomic level in multiple species of Hymenoptera. By summarizing the NUMT data obtained from whole genome scan in 35 insects, including the 11 fig wasp species in this study; four other Hymenoptera species; and 20 species from other orders of Diptera, Lepidoptera, and Coleoptera [8,9,11,22,40], we find that the average number, length, and proportion of NUMTs in the genome are all significantly higher in Hymenoptera than in other insects (Wilcoxon test, *p* < 0.05) (Figure 6). These results further support the previous report that NUMTs may be more common in Hymenoptera than in other insect genomes [11]. These NUMT-rich species are helpful for us to explore the biological issues behind the distribution and evolution of NUMTs.

We detect no correlation between the length, number, and the percentage of NUMTs and genome size in 11 fig wasps, and a similar pattern has been detected in the NUMTs of 13 eukaryotes [41]. In addition, in birds, a group whose genome size and structure are considered to be very stable, the number of NUMTs in different clades varies by hundreds of times [25]. On the contrary, the data from 85 genomes (from fungi and plants to mammals) have revealed a strong correlation between genome size and total NUMT length [22]. Therefore, when analyzing a large number of species including multiple taxa, NUMT content may be related to genome size; for a small number of species in one group, the correlation between the genome size and NUMT content may be unreliable.

In the 11 fig wasp genomes, the content and distribution of NUMTs were species- and lineage-specific. In general, the four species *W. pumilae*, *P. tridentata*, *D. vasculosae*, and *Sycobia* sp.2 showed a burst trend in terms of the number and length of NUMTs. Among them, *P. tridentata* and *A. bakeri*, within the same phylogenetic lineage, had the highest proportion of “duplicate NUMTs”. For the other lineage, *W. pumilae* and *D. vasculosae* had the largest number of “insertion type” NUMTs and “complex NUMTs”, also having a large number of “duplicate type” NUMTs. There are many factors influencing the burst of NUMTs. First of all, we examined the best-characterized *YME1* gene related to mtDNA escape, and the results of the RELAX algorithm [38] showed no evidence that the relaxation of purifying selection pressure acted on these four species to cause NUMTs to burst. Second, considering that *Sycobia* sp.2 and *P. tridentata* had the largest and the second-largest genome among all the studied fig wasp species, their large genome sizes may be an important factor in their abundant NUMT contents. On the one hand, a larger genome size means a higher chance of DSBs occurring, which will increase the opportunities for the insertion of NUMTs. In fact, previous studies have shown that the number of spontaneous DSBs increases after the yeast chromosome multiplication [42]. On the other hand, the loss rate of NUMTs in a large genome is also lower [43]. Thus, our results on fig wasps confirmed the effect of genome size on the distribution and burst of NUMTs.

We analyzed the insertion sites of NUMTs in the nuclear genome and found that NUMTs in all the studied fig wasp species tended to exist in high-AT-content, TE-rich, and noncoding regions. These results indicate that these regions are damage-prone regions of the nuclear genomes, and DSBs are easily produced under the action of internal and external factors, which further supports the correlation between DSBs and the insertion of NUMTs proposed by previous studies [22]. All the NUMTs of these fig wasp species were located in the intergenic and intronic regions, while no NUMTs in the gene coding regions were found. It may be that the NUMTs inserted into the noncoding regions are neutral, while the NUMTs inserted into the coding regions are usually harmful and will be deleted by purification selection [44]. In some rare cases reported in humans, NUMTs inserted into coding regions of genes can produce genetic diseases or altered phenotypes [45,46]. In summary, the above results indicate that different regional characteristics in the nuclear genome will affect the insertion and elimination of mtDNA.

When analyzing the length of NUMTs and the similarity with mtDNA, we found that the sequences of NUMTs produced by recent transfers were longer, and older (more diverged) NUMTs were shorter. These results indicated that mtDNA was initially integrated into the nuclear genome in the form of large fragments, which gradually became small fragments in the evolutionary process. We also found the existence of some “complex NUMTs” originating from the same initial insertion event of mtDNA, further proving that these NUMTs were generated by fragmentation. Moreover, we found that there was a very high proportion of “complex NUMTs” with TEs inside or around them, indicating that the fragmentation of NUMTs may be caused by the insertion of other sequences, especially TEs. In addition, we found that TEs also existed around most of the “duplicate NUMTs”; considering that NUMTs have no self-replication or transposition mechanism, they are expected to occur in tandems or be involved in duplication of larger segments in the genome [30], which suggests that the duplication of NUMTs is also closely related to TEs. For example, compared with *A. bakeri*, the burst of “duplicate NUMTs” in *P. tridentata* may be related to the abundance of Gypsy in its genome. In short, TEs are an important factor in the fragmentation and duplication events and thus the species-specific burst of NUMTs in the nuclear genome, and our results based on the NUMs data of fig wasp species support Michalovova’s hypothesis that the evolutionary fates of NUMTs in the genome are related to TEs [32].

Our study also found that there is still some burst of NUMTs that cannot be fully explained. For example, in the lineage of *D. vasculosae* and *W. pumilae*, although the genomic environment (especially TEs) can lead to the duplication and fragmentation of NUMTs, TEs cannot fully explain the burst of NUMTs in this lineage, as more than half of “duplicate type” NUMTs in both species have no TEs on the flanking sequences, and more than half of “complex NUMTs” in *D. vasculosae* have no TEs inside or on the flanking sequences. Moreover, the possible causes of the NUMT burst cannot be found from the perspective of genome size and mtDNA escape. Therefore, we speculate that the burst may be due to the relaxation of the purification selection of the genomes during a certain period of evolution, thereby retaining more NUMTs. In the future, adding more closely related species for research may provide us with more evidence about the cause of NUMT burst.

## 5. Conclusions

As a group of Hymenoptera, fig wasps are rich in NUMTs, and the content and distribution of NUMTs show species- and lineage-specificity. This specificity may originate from the influence of various genomic environments. The distribution characteristics of NUMTs in different species are shaped by the influence of (1) genome size on the capacity of NUMTs, (2) damage-prone regions on the selection of NUMT insertion positions, and (3) the vicinity of TEs on the fragmentation and duplication and the subsequent burst of NUMTs. Previous studies of NUMTs are often limited by the number of species, or the paucity of NUMTs in the selected taxa, or the lack of genome data, so only a part of the NUMT production process or one or two influencing factors can be discussed. In this study, multiple species of fig wasps from the Hymenoptera with abundant NUMTs are used to study the species-specific distribution pattern of NUMTs, so as to study the reasons and dynamics, at the genomic level. This study can provide a basis for further extensive and in-depth analysis of NUMTs.

## Figures and Tables

**Table 1:** The insertion sites of NUMTs in the fig wasp genomes.

Species	Intergenic Regions		Intronic Regions	
	Number ^1^	Proportion (%) ^2^	Number (gene) ^3^	Proportion (%) ^2^
*Dolichori vasculosae* *Wiebesia pumilae* *Eupristina koningsbergeri* *Platyscapa corneri* *Ceratosolen fusciceps* *Kradibia gibbosae* *Sycobia* sp.2 *Apocrypta bakeri* *Philotrypesis tridentata* *Sycophaga agreansis* *Sycophila* sp.2	443 522 148 105 157 99 278 124 582 56 68	75.085 83.121 67.580 69.079 77.723 64.286 67.371 77.019 94.175 51.376 61.818	147 (69) 106 (56) 71 (66) 47 (31) 45 (31) 55 (44) 139 (90) 37 (21) 36 (15) 53 (16) 42 (25)	24.915 16.879 32.420 30.921 22.277 35.714 32.629 22.981 5.825 48.624 38.182

^1^ The number of NUMTs in intergenic or intronic regions. ^2^ The proportion to the number of total NUMTs. ^3^ The number of genes involved.

## Supplementary Materials

The following are available online at https://www.mdpi.com/2075-4450/11/10/680/s1, Table S1: The amount of NUMTs in fig wasp genomes, Supplementary material: NUMT annotations.
