# Supplementary material for: Tracking the Distribution and Burst of Nuclear Mitochondrial DNA Sequences (NUMTs) in Fig Wasp Genomes
Source: Insects. 2020 Oct 7;11(10):680. doi: 10.3390/insects11100680 (PMC7600805; doi:10.3390/insects11100680)
Supplement: Supplementary file 1 [file insects-11-00680-s001.zip › Manuscript.docx]

Article

Tracking the distribution and burst of nuclear mitochondrial DNA sequences (*numts*) in fig wasp genomes

Jian-Xia Wang^1^, Jing Liu^1, †^, Yun-Heng Miao^2^, Da-Wei Huang^1, 2, ∗^, Jin-Hua Xiao^1, ∗^

^1^ Institute of Entomology, College of Life Sciences, Nankai University, Tianjin 300071, China.; [jianxiamail@163.com](mailto:jianxiamail@163.com) (J.-X. W.); [57506196@qq.com](mailto:57506196@qq.com) (J.L.)

2 Key Laboratory of Zoological Systematics and Evolution, Institute of Zoology, Chinese Academy of Sciences, Beijing 100101, China.; [fcbayernmyh@126.com](mailto:fcbayernmyh@126.com) (H.-Y.M.)

***** Correspondence: huangdw@ioz.ac.cn (D.-W.H.); xiaojh@nankai.edu.cn (J.-H.X.); Tel.: +86-139-1025-6670 (D.-W.H.); +86-185-2245-2108 (J.-H.X.)

^†^ These authors contributed equally to this work.

Received: date; Accepted: date; Published: date

**Simple Summary:** Nuclear mitochondrial DNA sequences (*numts*), which result from the insertion of exogenous mtDNA into the nuclear genome, are widely distributed in eukaryotes. But how *numts* are inserted into nuclear genome and their post-insertion fate remains a mystery. Previous studies suggested that Hymenoptera might be a group rich in *numts*, which would be helpful to study the biological issues of *numts*. We here select 11 species of fig wasps (Chalcidoidea, Hymenoptera) to analyze the distribution and evolution of *numts* at the genomic level. The results show that the distribution of *numts* were species- and lineage-specific. Furthermore, the genomic environment such as genome size, the damage-prone regions and the mode of TE dynamics, can determine the insertion and post-insertion fate of *numts*. Especially because of TEs, the fragmentation and duplication, and thus the burst of *numts* are common. This is a relatively comprehensive investigation of the specific distribution of *numts* and its influencing factors. Our study will help people to understand the evolution of exogenous fragments in the nuclear genome.

**Abstract:** Mitochondrial DNA sequences can be transferred into the nuclear genome, giving rise to *numts* (nuclear mitochondrial DNA sequences). *Numts* have been described in numerous eukaryotes. However, the studies on the distribution of *numts* and its influencing factors were still inadequate and even controversial. Previous studies suggested that Hymenoptera might be a group rich in *numts*, in which we selected 11 species of fig wasps (Chalcidoidea, Hymenoptera) to analyze the distribution and evolution of *numts* at the genomic level. The results showed that the contents of *numts* varied greatly in these species, and there existed bursts of *numts* in some species or lineages. Further detailed analyses showed that the large number of *numts* might be related to the large genomes; *numts* tended to be inserted into unstable regions of the genomes; and the inserted *numts* might also be affected by transposable elements (TEs) in the neighbors, leading to fragmentations and duplications, and then bursts of *numts*. In summary, our results suggest that a variety of genomic environmental factors can determine the insertion and post-insertion fate of *numts*, resulting in their species- or lineage-specific distribution patterns, and that studying the evolution of *numts* can provide good evidence and theoretical basis for exploring the dynamics of exogenous DNA entering into the nuclear genome.

**Keywords:** *numts*, mitochondrial DNA, transposable elements, evolution, fig wasps

1. Introduction

In eukaryotes, mitochondrial DNA sequences are frequently transferred into the nuclear genome, generating nuclear mitochondrial DNA sequences (*numts*) [1]. *Numts* may mislead barcoding, phylogenetic and phylogeographic inferences when they are regarded as true mitochondrial sequences [2]. However, considering that the mutation rate of nuclear DNA is lower than that of the mitochondrial genome, as nuclear DNA, *numts* are often used as molecular fossils to infer and calibrate speciation events [3-5]. Insect genomes have so far been reported to have very few or no copy of *numts*. For example, the *Anopheles gambiae* has none, *Drosophila melanogaster* has only few short *numts*, and *Bombyx mori* has dozens of *numts* [6-9]. However, in Hymenoptera, more than 1000 *numts* have been found in honeybees [10]; 195 *numts* have been found in *Nasonia vitripennis*, but considering that the *Nasonia* mitochondrial genome is only partially reported, there should be more *numts* [11]. So Hymenoptera probably have more *numts* than other insects.

It is generally believed that *numts* are inserted into double-strand breaks (DSBs) in the nuclear genome via a nonhomologous end joining (NHEJ) mechanism [12-14]. Previous studies on the integration sites of *numts* have found that although they are not randomly distributed in the genomes, their insertion sites are different in different species. *Numts* in human genome tend to be inserted into high-predicted DNA curvature and open chromatin regions, and often adjacent to AT oligomers [15,16]; the integration of *numts* in human genome is concentrated in intron regions [17] which is similar in honeybee [18]. However, in pig genome, most *numts* are located mainly in intergenic regions, and tend to be in regions of high GC content and near repetitive elements [19].

The number of *numts* varies among different species [20,21], but the factors are still unclear. The data on *numts* insertion frequency from 85 eukaryotic genomes revealed a strong correlation between genome size and *numt* contents, which implies that the rate of *numts* insertion might be limited by the frequency of DSBs [22]. In addition, mutation or inactivation of gene *YME1* in yeast and human can increase the escape rate of mtDNA, resulting in the burst of *numts* [14,23,24]; however, *numt* bursts were not associated with relaxed purifying selection on *YME1* in birds [25]. Generally speaking, *numts*, which are produced by the integration of exogenous DNA, the mtDNA, into the nuclear genome, will be fragmented and eliminated in the process of natural selection. However, in some cases, duplication and fragmentation events will occur after the insertion of *numts* into the genome [26,27], which is also the key reason for the burst of *numts* [28-30] ，and TEs may play an important role in it [17,30]. Based on the analysis results of *numts* in six plant genomes, Michalovova et al. proposed a model how TEs were involved in the evolution of *numts*: after new mtDNA sequences were integrated into the genome, the insertion of TEs made them fragmented, and TE-based recombination can result in deletion, replication, or movement of these *numts* fragments [31]. However, whether the hypothesis can be applied to more taxa remains to be verified.

In fact, the generation of *numts* includes the processes of the escape of mtDNA, the integration into the nuclear genome, and the dynamics of post-insertion [22], each of which will be affected by different or even multiple factors, and then produce different results. At present, the research on species-specific distribution of *numts* and its influencing factors is still in the exploratory stage. Most of the relevant reports only focus on a part of the process of *numts* production, or one or two influencing factors, and some results need to be verified by adding more taxa. In this study, the whole genome sequences data of 11 species of fig wasps (Hymenoptera, Chalcidoidea) completed in our lab were used to analyze the distribution and evolution of *numts*. We not only revealed the species- and lineage-specific patterns of the distribution and burst of *numts*, but also benefited from the sufficient amount of data, comprehensively deciphered the origins of these specific distribution patterns. This will help us to understand the evolution of exogenous fragments in the nuclear genome.

2. Materials and Methods

The whole nuclear genome and mitochondrial genome sequences of the 11 fig wasps were obtained from our lab. Date for the mitochondrial genome sequences of 11 fig wasp species were deposited in GenBank under the accession numbers: *Dolichoris vasculosae* (MT947596), Wiebesia pumilae (MT947601), *Eupristina koningsbergeri* (MT947597), *Platyscapa corneri* (MT947604), *Ceratosolen fusciceps* (MT916179), *Kradibia gibbosae* (MT947598), *Sycobia* sp.2 (MT947600), *Apocrypta bakeri* (MT906648), *Philotrypesis tridentata* (MT947602), *Sycophaga agreansis* (MT947599), *Sycophila* sp.2 (MT947603).

To identify *numts*, mtDNA was used as query for blastn against nuclear genome. The search was done using blast v2.10.0+ with e-value < 0.0001, while removing the hits less than 50 bp. The *numt* annotations are provided as Supplementary to this manuscript. The *numts* data of other insect species were obtained from previous literatures.

The genomic context of the detected *numts* was analyzed to identify (1) The AT contents (5 bp windows) of 250 bp upstream and downstream flanks of each *numt* and 50 bp at both ends of *numt*; (2) the position of insertion (intergenic, intronic, and coding regions). *Numts* and TEs density was calculated using 50 kb sliding windows by using Perl script. The number of TEs in the vicinity of *numts* (5 kb) was estimated using the RepeatMasker-4.1.0 software (available from: http://www. repeatmasker.org/).

Pairs of *numts* and their flanking DNA were compared to identify the “duplicate *numts*” arosed by DNA duplication. If one *numt* arosed from another by DNA duplication in the nucleus, homology between the *numts* should extend into the nuclear DNA that flanks them and the degree of similarity between the *numts* and between flanking DNA regions should be the same. The length and percentage similarity of *numts* and flanking DNA homology (if present) were determined using blastn.

In the identification of “complex *numts*”, every hits identified by blastn that fulfilled the next three criteria were considered to be part of one insertion event, and thus included in the list of “complex *numts*”: (1) *Numts* separated by < 10 kb of DNA of nonmitochondrial origin were considered as a cluster; (2) The *numts* in the same cluster had a very good synteny relationship with the corresponding mitochondrial region; (3) The direction of the *numts* sequence within the same cluster was consistent with the corresponding mitochondrial region.

3. Results

3.1. dentification of numts in the fig wasp genomes

We searched for *numts* in 11 fig wasp genomes by using blastn, and the results showed that the largest number of *numts* was present in *W. pumilae* (628 *numts*), followed by *P. tridentata* (618 *numts*), *D. vasculosae* (590 *numts*), and *Sycobia* sp.2 (426 *numts*), and the least was present in *S. agreansis* (109 *numts*). The longest total length of munts was present in *W. pumilae* (752,110 bp), followed by D. vacsulosae (671,621 bp), *P. tridentata* (374,545 bp), and *Sycobia* sp.2 (265,482 bp), and the shortest was present in *Sycophila* sp.2 (246,620 bp). In terms of the percentage of *numts* in the genome, the highest genome content of *numts* was 0.235% in both *W. pumilae* and D. vassulosae, followed by *P. tridentata* (0.094%) and *A. bakeri* (0.065%), and the lowest was in *Sycophila* sp.2 (0.016%) (Figure 1A, Table S1). Therefore, the number and length of *numts* in *W. pumilae*, *P. tridentata*, *D. vasculosae*, and *Sycobia* sp.2 all showed a burst trend. Among them, *W. pumilae* and *D. vasculosae* were in the same evolutionary lineage, and they had the longest length and highest genome content of *numts*. Therefore, it can be seen that the number and length of *numts* varied remarkably across different species, showing distribution of not only species-specificity but also lineage-specificity. We further carried out correlation analysis between the length, number, and the percentage of *numts* in the genome and genome size for the 11 fig wasps, and the spearman correlation coefficients were 0.39, 0.55, and 0.09, respectively, with no significant correlation (Wilcoxon test, P > 0.05). However, *Sycobia* sp.2, with the largest genome, and P. tridentate, with the second largest genome, were indeed rich in *numts* number and length.


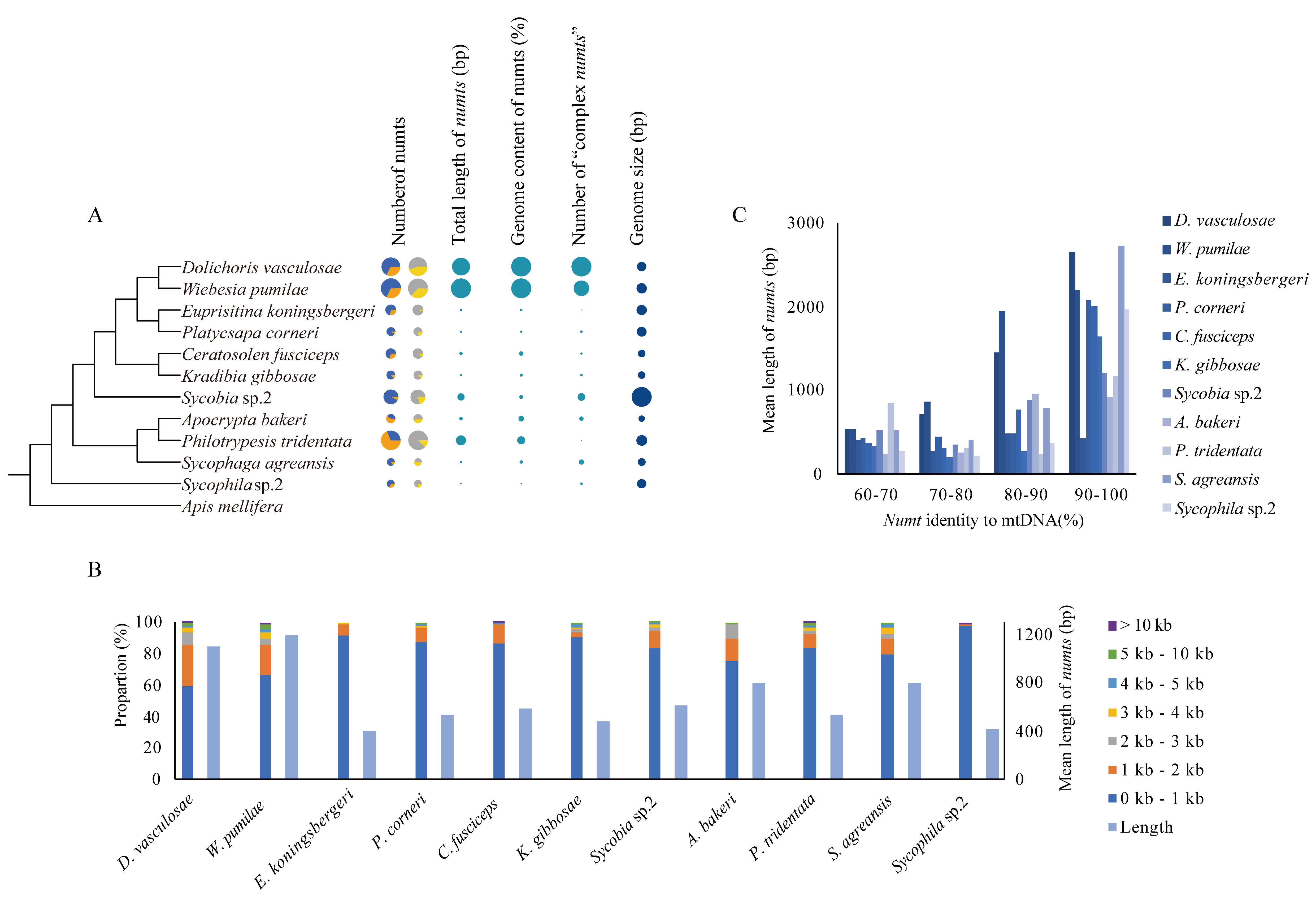


**Figure 1.** Distribution of *numts* in fig wasp genomes. (A) The amount of *numts* in the fig wasps. The phylogenetic tree on the left was referenced from our unpublished phylogenomic work, and the pie chart columns on the right display the statistical results of the *numts*. The size of the pie chart corresponds to the amount of data in each group. In the result group of “Number of *numts*”, the pie chart in the first column shows the content of “duplicate type” *numts* (orange) and “insertion type” *numt* (blue), while the second column shows the content of *numts* contained in “complex *numts*” (yellow), compared to others (grey). (B) The length distribution of *numts*. Each species had two columns, with the left one representing the proportion of *numts* in different length ranges (corresponding to values on the left vertical axis), and the right one representing the mean length of *numts* in the species (corresponding to the values on the right vertical axis). (C) The correlation between *numts* length and the sequence divergence of *numts* with their corresponding mtDNAs. For each species, the average lengths of *numts* of different ranges of similarity with their mtDNA are shown, and in general, the higher the similarity to the corresponding mtDNA, the longer the average length of *numt*.

In the process of *numts* generation, some of them arose directly from the insertion of mtDNA into nuclear genome (we named them “insertion type”), while some *numts* are produced by duplication of *numts* that have been inserted into the nuclear genome (we named them “duplicate type”). For these “duplicate type” *numts*, some nuclear DNA sequences on its both flanks are often included in their repetitive duplications and insertions; therefore, sequence similarity will extend to the flanking nuclear DNA sequences when sequence alignments were demonstrated [32], and based on this, we can identify those “duplicate type” *numts* (Figure 1A,Table S1). Among the studied 11 fig wasp species, the largest number of “duplicate type” *numts* was present in *P. tridentata* (425 *numts*), followed by *W. pumilae* (202 *numts*) and *D. vasculosae* (193 *numts*), and Platycsapa corneri (15 *numts*) was the least. The proportion of “duplicate type” *numts* in all *numts* varied among the species, with the largest proportion present in *P. tridentata* (68.77%) and *A. bakeri* (60.87%), followed by *D. vasculosae* (32.71%) and *W. pumilae* (32.17%), and the least was *Sycobia* sp.2 (7.04%). It can be seen that the distribution of “duplicate type” *numts* showed lineage-specificity: *P. tridentata* and *A. bakeri* had the highest proportion, *D. vasculosae* and *W. pumilae* were the second in number and proportion (Figure 1A, Table S1). For each species, we could get the number of “insertion type” *numts* after removing the “duplicate type” *numts* from the total *numts*. As to the comparison of the number of “insertion type” *numts*, *W. pumilae* (426 *numts*) had the largest number of “insertion type” *numts*, followed by *D. vasculosae* (397 *numts*), *Sycobia* sp.2 (396 *numts*), *P. tridentata* (193 *numts*), and the least was *A. bakeri* (63 *numts*). Therefore, species with more “insertion type” *numts* also showed lineage-specific characteristics (Figure 1A, Table S1).

We investigated the length distribution of *numts* and their similarity to mitochondrial genes, and the results showed that in all species, most identified *numts* were short fragments (mean lengths ranged from 299 bp for *Sycophila* sp.2 to 1,231 bp for *W. pumilae*), and many of them were shorter than 1,000 bp (58.5% in *D. vasculosae* to 98.2% in *Sycophila* sp.2) (Figure 1B). However, in some species, the longest *numts* were more than 10 kb in length, such as in *D. vasculosae* (12,107 bp), *W. pumilae* (12,250 bp), P. tridentate (14,100 bp), and *Sycophila* sp.2 (14,898 bp). We further compared the sequence similary between the *numts* and their corresponding original mtDNA, and the results showed that longer *numts* tended to be more similar to their corresponding mtDNA sequences (Figure 1C), implying their recent origin.

For each species, when we mapped all the *numts* to their corresponding mitochondrial genome, we found that *numts* might originate from any region of the mitochondrial genome (Figure 2A). In addition, we also noticed that some adjacent *numts*, which we called “complex *numts*”, may be derived from the fragmentation of the same “insertion type” *numt* (eg. in Figure 2B) [30], we used strict criteria to identify and count the number of “complex *numts*” in each species. As a result, 196 “complex *numts*” were identified in 11 fig wasps, with the numbers varied among different species: The largest number is 55 in *D. vasculosae*, and the least is in Euprisitina koningsbergeri with only two. In particular, in the lineage of *D. vasculosae* and *W. pumilae*, both of them had the largest number of *numts* and the largest number of “complex *numts*”, indicating that fragmentation contributed a lot of *numts* in their genomes (Figure 1A, Table S1).


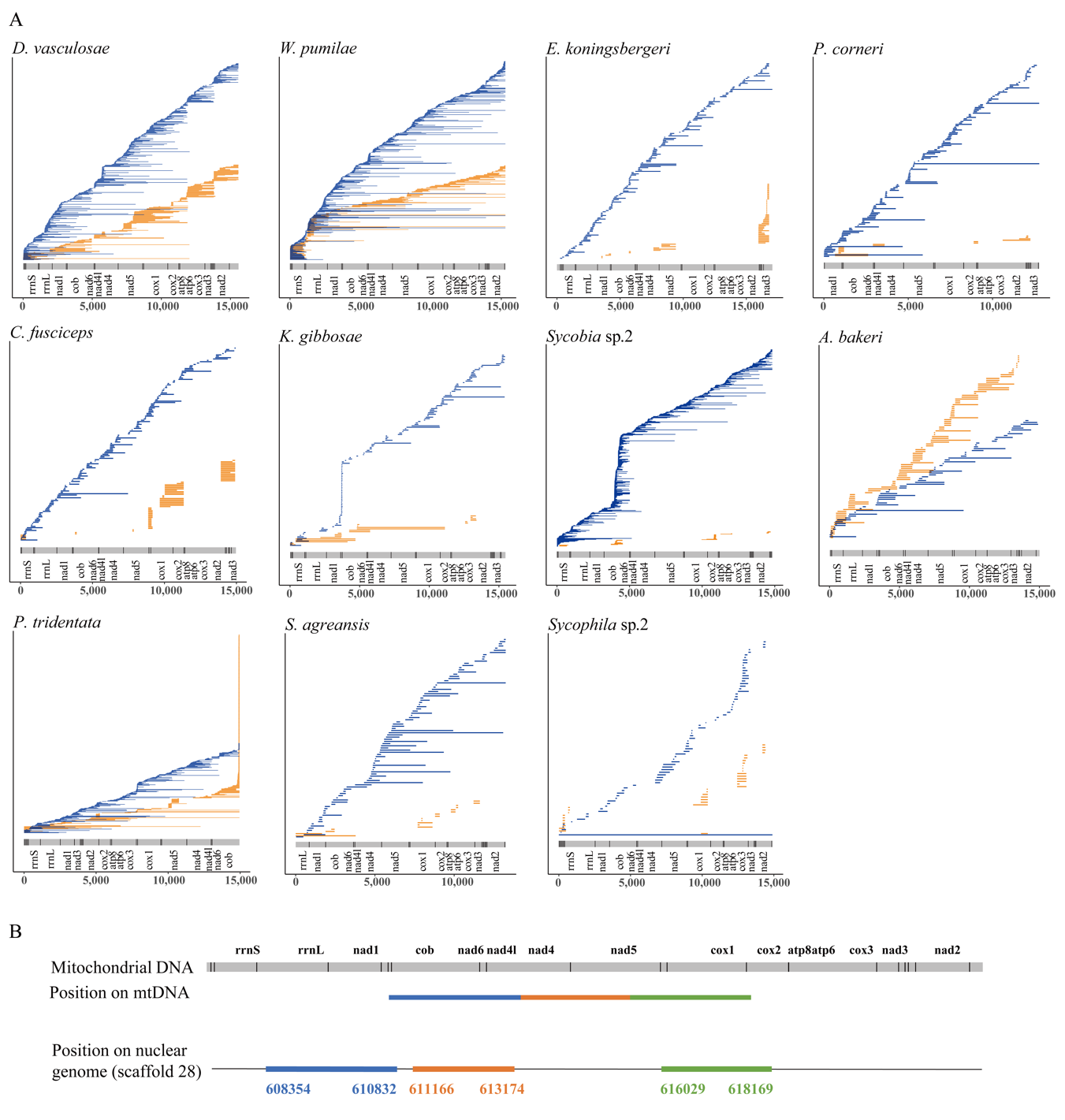


**Figure 2**. Mapping of *numts* and their mitochondrial genome regions (A) and a detailed display of one example of fragmentation event in the species of *A. bakeri* (B). (A) For each species, the *numts* were mapped onto their original positions on the mitochondrial genome (the length and genes of mitochondrial are shown in the bottom). The orange lines represent the “duplicate type” *numts* and the blue lines represent “insertion type” *numts*. (B) The picture showed a 9,815 bp long sequence containing three *numts* (in different colors) on scaffold 28 in the genome of *A. bakeri* (in the bottom), and the origin of the three *numts* on its mitochondrial genome (on the top). The numbers show the start and end positions of different *numts* on the scaffold. On the mitochondrial genome, transfer RNA genes are indicated with vertical lines; acronyms stand for: NADH dehydrogenase subunits 1 to 6 (nad1-6), cytochrome c oxidase subunits I to III (cox1-cox3), ATP synthase 6 and 8 (atp6 and atp8), cytochrome b (cob), 16S and 12S rRNA (rrnL and rrnS).

3.2. Characteristics of numt insertion regions in the genomes

In order to explain the specific distribution of *numts* and its influencing factors, we analyzed the genomic environment where *numts* were inserted. First, the AT content of *numts* and their flanking sequences were analyzed. The results showed a high AT content in flanking regions (±250 bp) of *numts* in all species (Figure 3A). In each species, the 10 bp flanking regions of the *numt* sequences showed a significant higher AT content than the average AT content in the scaffold they are located (Wilcoxon test, P < 0.01) (Figure 3B).


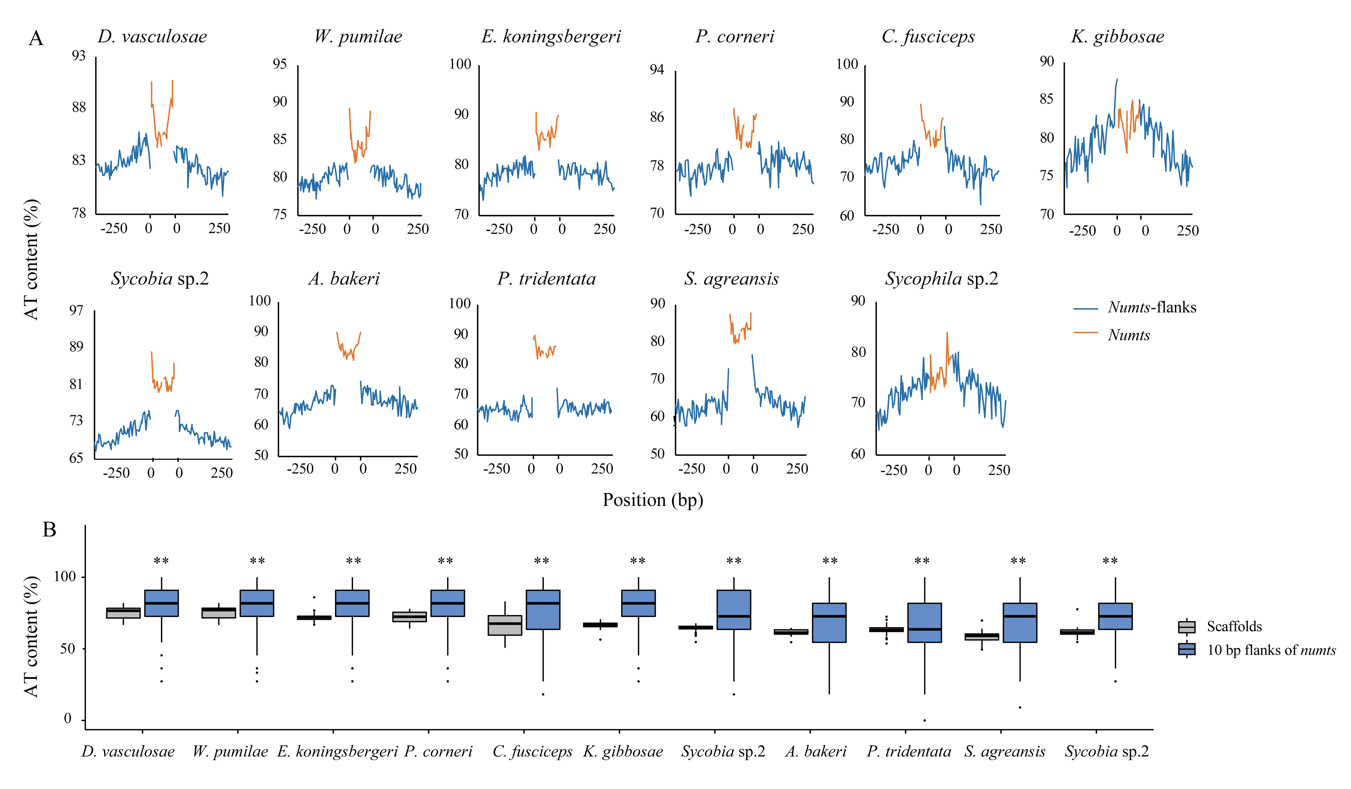


Figure 3. Statistic of the mean AT content in *numts* flanking sequences. (A) For each species, the mean AT content (5 bp windows) of 250 bp flanks of all *numts* (blue lines) and 50 bp at both ends of all *numts* (orange lines) are displayed. The horizontal axis gives the positions of *numts* and *numt* flanking regions in genome. The vertical axis gives the mean AT content at each position. (B) Comparison of mean AT content in the 10 bp flanking regions of all *numts* to the mean AT content of all scaffolds containing *numts* in the genome. **P < 0.01, Wilcoxon test.

When surveyed the position information of *numts* in the nuclear genome, we found that more than half of the *numts* of each fig wasp species were located in intergenic regions. Among them, the proportion was as high as 94.2% in *P. tridentata*, while it was only 51.4% in S. argeansis, which was the lowest. Except the *numts* in intergenic regions, the rest of the *numts* in each species were located in the intronic regions within genes. In the *D. vasculosae* genome, a total of 147 out of the detected 490 *numts* were located within 69 different annotated genes，which was the largest number of *numts* located in the intronic regions in these species. The least was in *P. tridentata* (36 *numts*). No *numts* were found in the protein coding regions in all of the species (Table 1).

**Table 1.** The insertion sites of *numts* in the fig wasp genomes.

| **Species** |  | **Intergenic regions** | |  | **Intronic regions** | |
| --- | --- | --- | --- | --- | --- | --- |
|  |  | **Number^1^** | P**roportion (%)^2^** |  | **Numbera (gene^3^)** | **Proportion (%)^2^** |
| *D. vasculosae*  *W. pumilae*  *E. koningsbergeri*  *P. corneri*  *C. fusciceps*  *K. gibbosae*  *Sycobia* sp.2  *A. bakeri*  *P. tridentata*  *S. agreansis*  *Sycophila* sp.2 |  | 443  522  148  105  157  99  278  124  582  56  68 | 75.085  83.121  67.580  69.079  77.723  64.286  67.371  77.019  94.175  51.376  61.818 |  | 147 (69)  106 (56)  71 (66)  47 (31)  45 (31)  55 (44)  139 (90)  37(21)  36(15)  53(16)  42(25) | 24.915  16.879  32.420  30.921  22.277  35.714  32.629  22.981  5.825  48.624  38.182 |

^1^The number of *numts* in intergenic or intronic regions. ^2^The proportion to the number of total *numts*. ^3^The number of genes involved.

We also studied the correlation between the distributions of *numts* and TEs. The distribution density of TEs and *numts* were surveyed based on 50 kb windows, with TEs including DNA transposon, long interspersed nuclear elements (LINE)，short interspersed nuclear elements (SINE), rolling cycle (RC), and long terminal repeats (LTR). As was shown in Figure 5, in most species, the pattern of *numts* distribution was similar to TEs, that is, where the density of *numts* was high, the density of TEs was also high (Figure 4).


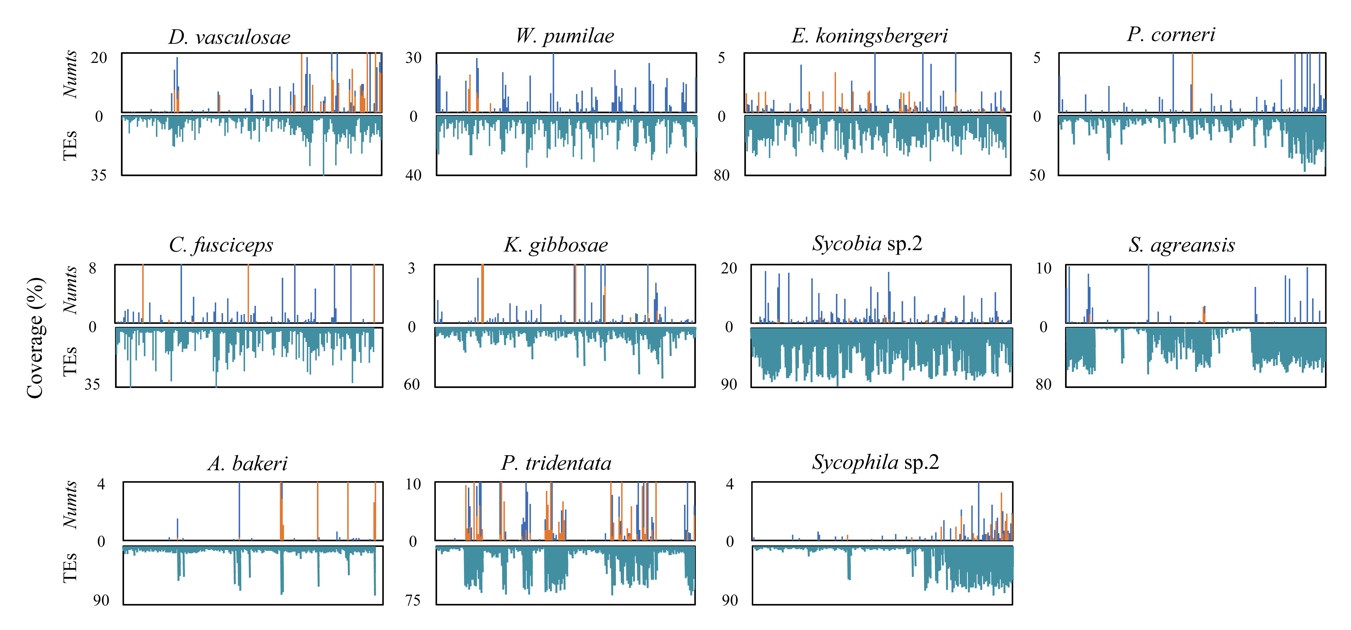


Figure 4. The density distribution of *numts* and TEs on the genome. The results of each species are shown in separate panels, with the distribution of “insertion type” (as labeled in blue color) and “duplicate type” (as labeled in orange color) *numts* on top of the panel and that of total TEs (including DNA transposon, LINE, SINE, RC, and LTR) at bottom. The sliding window size was set to 50 kb. The horizontal axis represents position of the windows in the nuclear genome, and the vertical axis represents the numbers of *numts* or TEs in the sliding windows.

3.3. Relationship between duplication/fragmentation events of numts and TEs

As mentioned above, there were “duplicate type” *numts* and “complex *numts*” in all fig wasp species. Coupled with the similar density distribution of *numts* and TEs in genomes, we further studied the distribution of TEs near “duplicate type” *numts* and “complex *numts*”, in order to obtain more evidence about the correlation between *numts* and TEs. By using RepeatMasker software, we analyzed whether there was TEs on the flanks (< 5 kb) of “duplicate type” *numts*. As a result, in the three species of *A. bakeri*, *S. agreansis* and *Sycophila* sp.2, TEs existed on the flanks of all “duplicate type” *numts* (Figure 5A), while in the other species, the proportion of “duplicate type” *numts* containing TEs on the flanking sequences varied, of which the least was in *D. vasculosae* (30%). Further, we counted the TE types closest to “duplicate type” *numts*. The result showed that the largest proportion of TEs was Gypsy in *W. pumilae*, *A. bakeri* and *P. tridentata*, accounting for 46%, 44%, and 36%, respectively. In *D. vasculosae*, Jockey had the largest proportion (59%) of TEs closest to “duplicate type” *numts* (Figure 5B).


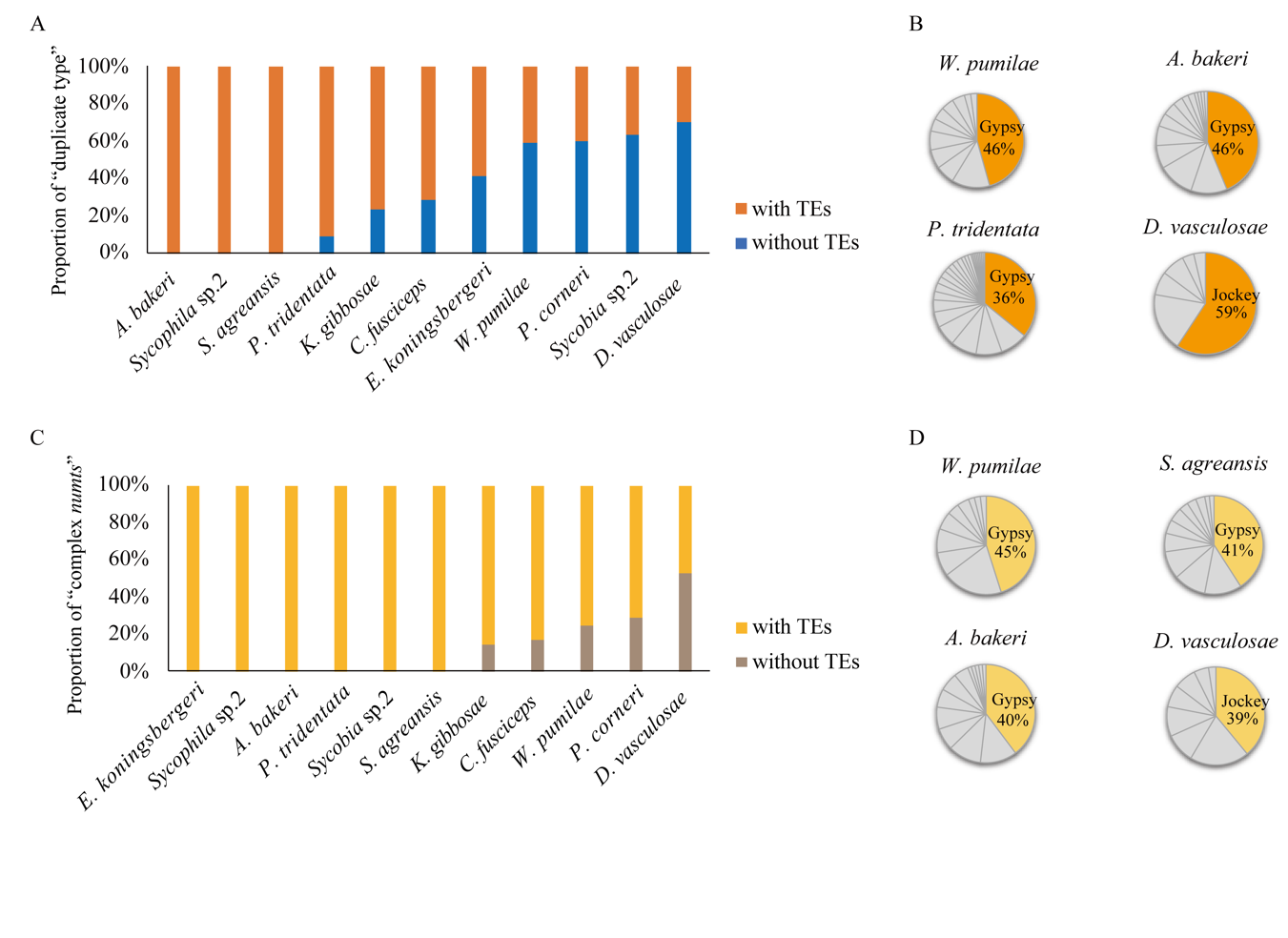


Figure 5. Correlation of TEs with “duplicate type” *numts* and “complex *numts*”. (A) The proportion of “duplicate type” *numts* with or without TEs on the flanking sequences. (B) Pie charts showing the content of different TE superfamilies in flankings of “duplicate type” *numts* in the four species of *W. pumilae*, *A. bakeri*, *P. tridentata*, and *D. vasculosae*. The color areas represent the TEs type with the highest proportion in each species. (C) The proportion of “complex *numts*” with or without TEs on the interior and flanking sequences. (D) Pie charts showing the content of different TE superfamilies in flankings or interior region of “complex *numts*” in the four species of *W. pumilae*, S. agraensis, *A. bakeri*, and *D. vasculosae*. The color areas represent TEs type with the highest proportion in each species. In the panel (B) and (D), the results of TEs type proportions of only four species are shown, because the amount of TEs of the other species is small, or the proportions of different TEs types are very similar.

As mentioned above, *P. tridentata* and *A. bakeri*, the two species of Pteromalidae, had the highest proportion of “duplicate type” *numts* in all species, but the number of “duplicate type” *numts* in *A. bakeri* (98 *numts*) was much less than that in *P. tridentata* (425 *numts*) (Figure 1A, Table S1). Further analysis showed that the *numts* burst in *P. tridentata* was mainly caused by a large number of duplications of *numts* corresponding to the 14885-14950 bp region of its mitochondrial genome (Figure 2A). In terms of the TEs in the flanking sequences of “duplicate type” *numts*, more than 90% of the *numts* flanks contained TEs in both species (Figure 5A), and the number of LTR/Gypsy was the largest (Figure 5B). Combined that the Gypsy sequences accounted 6.866% and 1.290%, of the total genome of *P. tridentata* and *A. bakeri*, respectively (the data was referenced from our unpublished phylogenomic work), it was speculated that the burst of “duplicate type” *numts* in *P. tridentata* was closely related to the abundance of Gypsy in its genome.

When we searched for TEs within and around “complex *numts*”, we found that there were six species (*A. bakeri*, *E. koningsbergeri*, *P. tridentata*, *Sycobia* sp.2, *Sycophila* sp.2, and S. agraensis), in which all of the “complex *numts*” had TEs inside or around, and in the remaining species more than 70% of the “complex *numts*” had TEs inside or around, except for *D. vasculosae*, with only 47% (Figure 5C). When we surveyed the type of these TEs, the results showed that the species with the largest proportion of Gypsy were *W. pumilae* (45%), S. agraensis (41%), and *A. bakeri* (40%), while *D. vasculosae* had the largest proportion of Jockey (39%) (Figure 5D).

4. Discussion

In this study, 11 fig wasp species were selected to analyze the number and distribution of *numts* in their genomes. This is the first detailed and systematic comparative study of the number and distribution of *numts* at the genomic level in multiple species of Hymenoptera. By summarizing the *numts* data obtained from whole genome scan in 35 insects, including the 11 fig wasp species in this study, four others Hymenoptera, and 20 species from other orders of Diptera, Lepidoptera, and Coleoptera [8,9,11,22,33], we found that the average number, length, and proportion of *numts* in the genome were all significant higher in Hymenoptera than in other insects (Wilcoxon test, P < 0.05) (Figure 6). These results further support the previous report that *numts* may be more common in Hymenoptera than in other insect genomes [11]. These *numts*-rich species are helpful for us to explore the biological issues behind the distribution and evolution of *numts*.

**
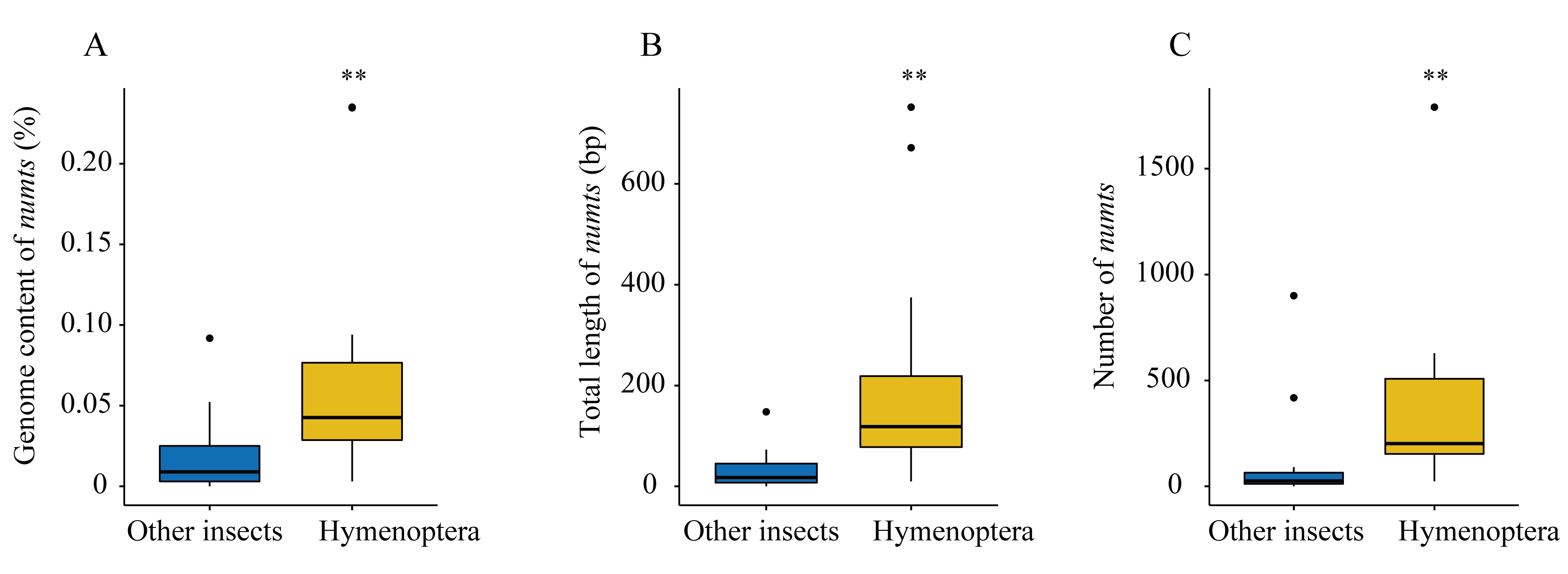
**

Figure 6. Comparison of *numts* in Hymenoptera and other insects (Diptera, Lepidoptera, and Coleoptera). The content of *numts* in the genomes (A), the total length of *numts* (B), and the number of *numts* (C) are compared. **P < 0.01, Wilcoxon test.

In the 11 fig wasp genomes, the content and distribution of *numts* were species- and lineage-specific. In general, the four species of *W. pumilae*, *P. tridentata*, *D. vasculosae*, and *Sycobia* sp.2 showed a burst trend in terms of the number and length of *numts*. Among them, P. tridentate and *A. bakeri*, within the same phylogenetic lineage, had the highest proportion of “duplicate *numts*”. While for the other lineage of *W. pumilae* and D. vassulosae, they had the largest number of “insertion type” *numts* and “complex *numts*”, and also a large number of “duplicate type” *numts*. There are many factors influencing the burst of *numts*. First of all, we examined the best-characterized *YME1* gene related to mtDNA escape, and the results of RELAX algorithm [34] showed no evidence that the relaxation of purifying selection pressure acted on these four species to cause *numts* to burst. Second, considering that *Sycobia* sp.2 and *P. tridentata* had the largest and the second largest genome among all the studied fig wasp species, their large genome sizes may be an important factor in their abundant *numts* contents. On the one hand, the larger the genome is, the more chances of DSBs will occur, which will increase the opportunities for the insertion of *numts*. In fact, previous studies have shown that the number of spontaneous DSBs increases after the yeast chromosome multiplication [35]. On the other hand, the loss rate of *numts* in large genome is also lower [36]. Thus, our results on fig wasps confirmed the effect of genome size on the distribution and burst of *numts*.

We analyzed the insertion sites of *numts* in the nuclear genome, and found that *numts* in all the studied fig wasp species tended to exist in high AT content, TEs rich and non-coding regions. These results indicate that these regions are damage-prone regions of the nuclear genomes, and DSBs are easily produced under the action of internal and external factors, which further supports the correlation between DSBs and the insertion of *numts* proposed by previous studies [22]. All the *numts* of these fig wasp species were located in the intergenic and intronic regions, while no *numts* in the gene coding regions were found. It may be that the *numts* inserted into the noncoding regions are neutral, while the *numts* inserted into the coding regions are usually harmful and will be deleted by purification selection [37]. In some rare cases reported in humans, *numts* inserted into coding regions of genes can produce genetic diseases or altered phenotypes [38,39]. In summary, the above results indicate that different regional characteristics in the nuclear genome will affect the insertion and elimination of mtDNA.

When analyzing the length of *numts* and the similarity with mtDNA, we found that the sequence of *numts* produced by recent transfers was longer, and older (more diverged) *numts* were shorter. These results indicated that mtDNA was initially integrated into the nuclear genome in the form of large fragments, which gradually became small fragments in the evolutionary process. We also found the existence of some “complex *numts*” originated from the same initial insertion event of mtDNA, further proving that these *numts* were generated by fragmentation. Moreover, we found that there were a very high proportion of “complex *numts*” having TEs inside or around, indicating that the fragmentation of *numts* may be caused by the insertion of other sequences, especially TEs. In addition, we found that TEs also existed around most of the “duplicate *numts*”; considering that *numts* have no self-replication or transposition mechanism, they are expected to occur in tandems or be involved in duplication of larger segments in the genome [30], which suggesting that the duplication of *numts* is also closely related to TEs. For example, compared with *A. bakeri*, the burst of “duplicate *numts*” in *P. tridentata* may be related to the abundance of Gypsy in its genome. In short, TEs are an important factor in the fragmentation and duplication events and thus species-specific burst of *numts* in the nuclear genome, and our results based on the *numts* data of fig wasp species support Michalovova’s hypothesis that the evolutionary fates of *numts* in the genome are related to TEs [31].

Our study also found that there is still some burst of *numts* that cannot be fully explained. For example, in the lineage of *D. vasculosae* and *W. pumilae*, although the genomic environment (especially TEs) can lead to the duplication and fragmentation of *numts*, TEs cannot fully explain the burst of *numts* in this lineage, as more than half of “duplicate type” *numts* in both species have no TEs on the flanking sequences, and more than half of “complex *numts*” in *D. vasculosae* have no TEs inside or on the flanking sequences. Moreover, the possible causes of the *numts* burst cannot be found from the perspective of genome size and mtDNA escape. Therefore, we speculate that the burst may be due to the relaxation of the purification selection of the genomes during a certain period of evolution, thereby retaining more *numts*. In the future, adding more closely related species for research may provide us with more evidence about the cause of *numts* burst.

5. Conclusions

As a group of Hymenoptera, fig wasps are rich in *numts*, and the content and distribution of *numts* show species- and lineage-specificity. This specificity may originate from the influence of various genomic environments. The distribution characteristics of *numts* in different species are shaped by the influence of 1) genome size on the capacity of *numts*, 2) damage-prone regions on the selection of *numts* insertion position, and 3) the vicinity of TEs influences the fragmentation and duplication, and the subsequent burst of *numts*. Previous studies of *numts* are often limited by the number of species, or the paucity of *numts* in the selected taxa, or the lack of genome data, so only a part of the *numts* production process or one or two influencing factors can be discussed. In this study, multiple species of fig wasps from the Hymenoptera with abundant *numts* are used to study the species-specific distribution pattern of *numts*, so as to the reasons and dynamics, at the genomic level. This study can provide a basis for the further extensive and in-depth analysis of *numts*.

**Supplementary Materials:** The following are available online at www.mdpi.com/xxx/s1, Table S1: The amount of *numts* in fig wasp genomes, Supplementary material: *Numt* annotations.

**Author Contributions:** DWH and JHX conceived the project; JXW and YHM performed experiments and analyzed the data. JXW wrote the manuscript, with input from JHX and JL. All authors reviewed the manuscript.

**Funding:** This work was supported by the National Natural Science Foundation of China (Nos of 31830084, 31970440 & 31672336), and also supported by the construction funds for the “Double First-Class” initiative for Nankai University (Nos. 96172158, 96173250 & 91822294).

**Acknowledgments:** We wish to thank our colleagues from the Naikai University and Chinese Academy of Sciences, especially the Institute of Entomology, Baojie Du, Yi Zhou and Zhaozhe Xin.

**Conflicts of Interest:** The authors declare no conflict of interest.

References

1. Lopez, J.V.; Yuhki, N.; Masuda, R.; Modi, W.; O'Brien, S.J. Numt, a recent transfer and tandem amplification of mitochondrial DNA to the nuclear genome of the domestic cat. J. Mol. Evol. 1994, 39, 174-190, doi:10.1007/bf00163806.
2. Song, H.; Buhay, J.E.; Whiting, M.F.; Crandall, K.A. Many species in one: DNA barcoding overestimates the number of species when nuclear mitochondrial pseudogenes are coamplified. Proc Natl Acad Sci U S A 2008, 105, 13486-13491, doi:10.1073/pnas.0803076105.
3. Zischler, H.; Geisert, H.; von Haeseler, A.; Pääbo, S. A nuclear 'fossil' of the mitochondrial D-loop and the origin of modern humans. Nature 1995, 378, 489-492, doi:10.1038/378489a0.
4. Perna, N.T.; Kocher, T.D. Mitochondrial DNA: molecular fossils in the nucleus. Curr Biol 1996, 6, 128-129, doi:10.1016/s0960-9822(02)00441-4.
5. Soto-Calderón, I.D.; Clark, N.J.; Wildschutte, J.V.; DiMattio, K.; Jensen-Seaman, M.I.; Anthony, N.M. Identification of species-specific nuclear insertions of mitochondrial DNA (numts) in gorillas and their potential as population genetic markers. Mol Phylogenet Evol 2014, 81, 61-70, doi: 10.1016/j.ympev.2014.08.018.
6. Richly, E.; Leister, D. NUPTs in sequenced eukaryotes and their genomic organization in relation to NUMTs. Mol Biol Evol 2004, 21, 1972-1980, doi:10.1093/molbev/msh210.
7. Behura, S.K.; Lobo, N.F.; Haas, B.; deBruyn, B.; Lovin, D.D.; Shumway, M.F.; Puiu, D.; Romero-Severson, J.; Nene, V.; Severson, D.W. Complete sequences of mitochondria genomes of Aedes aegypti and Culex quinquefasciatus and comparative analysis of mitochondrial DNA fragments inserted in the nuclear genomes. Insect Biochem Mol Biol 2011, 41, 770-777, doi: 10.1016/j.ibmb.2011.05.006.
8. Lämmermann, K.; Vogel, H.; Traut, W. The mitochondrial genome of the Mediterranean flour moth, Ephestia kuehniella (Lepidoptera: Pyralidae), and identification of invading mitochondrial sequences (numts) in the W chromosome. Eur. J. Entomol. 2016, 113, 482-488, doi:10.14411/eje.2016.063.
9. Rogers, H.H.; Griffiths-Jones, S. Mitochondrial pseudogenes in the nuclear genomes of Drosophila. PLoS ONE 2012, 7, e32593, doi: 10.1371/journal.pone.0032593.
10. Pamilo, P.; Viljakainen, L.; Vihavainen, A. Exceptionally high density of NUMTs in the honeybee genome. Mol Biol Evol 2007, 24, 1340-1346, doi:10.1093/molbev/msm055.
11. Viljakainen, L.; Oliveira, D.C.; Werren, J.H.; Behura, S.K. Transfers of mitochondrial DNA to the nuclear genome in the wasp Nasonia vitripennis. Insect Mol Biol 2010, 19 Suppl 1, 27-35, doi:10.1111/j.1365-2583.2009.00932. x.
12. Hazkani-Covo, E.; Covo, S. Numt-mediated double-strand break repair mitigates deletions during primate genome evolution. PLoS Genet 2008, 4, e1000237, doi: 10.1371/journal.pgen.1000237.
13. Jensen-Seaman, M.I.; Wildschutte, J.H.; Soto-Calderón, I.D.; Anthony, N.M. A comparative approach shows differences in patterns of numt insertion during hominoid evolution. J. Mol. Evol. 2009, 68, 688-699, doi:10.1007/s00239-009-9243-4.
14. Shafer, K.S.; Hanekamp, T.; White, K.H.; Thorsness, P.E. Mechanisms of mitochondrial DNA escape to the nucleus in the yeast Saccharomyces cerevisiae. Curr. Genet. 1999, 36, 183-194, doi:10.1007/s002940050489.
15. Dayama, G.; Emery, S.B.; Kidd, J.M.; Mills, R.E. The genomic landscape of polymorphic human nuclear mitochondrial insertions. Nucleic Acids Res. 2014, 42, 12640-12649, doi:10.1093/nar/gku1038.
16. Tsuji, J.; Frith, M.C.; Tomii, K.; Horton, P. Mammalian NUMT insertion is non-random. Nucleic Acids Res. 2012, 40, 9073-9088, doi:10.1093/nar/gks424.
17. Ricchetti, M.; Tekaia, F.; Dujon, B. Continued colonization of the human genome by mitochondrial DNA. PLoS Biol 2004, 2, E273, doi: 10.1371/journal.pbio.0020273.
18. Behura, S.K. Analysis of nuclear copies of mitochondrial sequences in honeybee (Apis mellifera) genome. Mol Biol Evol 2007, 24, 1492-1505, doi:10.1093/molbev/msm068.
19. Schiavo, G.; Hoffmann, O.I.; Ribani, A.; Utzeri, V.J.; Ghionda, M.C.; Bertolini, F.; Geraci, C.; Bovo, S.; Fontanesi, L. A genomic landscape of mitochondrial DNA insertions in the pig nuclear genome provides evolutionary signatures of interspecies admixture. DNA Res 2017, 24, 487-498, doi:10.1093/dnares/dsx019.
20. Calabrese, F.M.; Balacco, D.L.; Preste, R.; Diroma, M.A.; Forino, R.; Ventura, M.; Attimonelli, M. NumtS colonization in mammalian genomes. Sci Rep 2017, 7, 16357, doi:10.1038/s41598-017-16750-2.
21. Nacer, D.F.; Raposo do Amaral, F. Striking pseudogenization in avian phylogenetics: Numts are large and common in falcons. Mol Phylogenet Evol 2017, 115, 1-6, doi: 10.1016/j.ympev.2017.07.002.
22. Hazkani-Covo, E.; Zeller, R.M.; Martin, W. Molecular poltergeists: mitochondrial DNA copies (numts) in sequenced nuclear genomes. PLoS Genet 2010, 6, e1000834, doi: 10.1371/journal.pgen.1000834.
23. Portugez, S.; Martin, W.F.; Hazkani-Covo, E. Mosaic mitochondrial-plastid insertions into the nuclear genome show evidence of both non-homologous end joining and homologous recombination. BMC Evol Biol 2018, 18, 162, doi:10.1186/s12862-018-1279-x.
24. Srinivasainagendra, V.; Sandel, M.W.; Singh, B.; Sundaresan, A.; Mooga, V.P.; Bajpai, P.; Tiwari, H.K.; Singh, K.K. Migration of mitochondrial DNA in the nuclear genome of colorectal adenocarcinoma. Genome Med 2017, 9, 31, doi:10.1186/s13073-017-0420-6.
25. Liang, B.; Wang, N.; Li, N.; Kimball, R.T.; Braun, E.L. Comparative Genomics Reveals a Burst of Homoplasy-Free Numt Insertions. Mol Biol Evol 2018, 35, 2060-2064, doi:10.1093/molbev/msy112.
26. Collura, R.V.; Stewart, C.B. Insertions and duplications of mtDNA in the nuclear genomes of Old World monkeys and hominoids. Nature 1995, 378, 485-489, doi:10.1038/378485a0.
27. Hazkani-Covo, E.; Sorek, R.; Graur, D. Evolutionary dynamics of large numts in the human genome: rarity of independent insertions and abundance of post-insertion duplications. J. Mol. Evol. 2003, 56, 169-174, doi:10.1007/s00239-002-2390-5.
28. Antunes, A.; Pontius, J.; Ramos, M.J.; O'Brien, S.J.; Johnson, W.E. Mitochondrial introgressions into the nuclear genome of the domestic cat. J Hered 2007, 98, 414-420, doi:10.1093/jhered/esm062.
29. Erpenbeck, D.; Voigt, O.; Adamski, M.; Woodcroft, B.J.; Hooper, J.N.; Wörheide, G.; Degnan, B.M. NUMTs in the sponge genome reveal conserved transposition mechanisms in metazoans. Mol Biol Evol 2011, 28, 1-5, doi:10.1093/molbev/msq217.
30. Hazkani-Covo, E.; Martin, W.F. Quantifying the Number of Independent Organelle DNA Insertions in Genome Evolution and Human Health. Genome Biol Evol 2017, 9, 1190-1203, doi:10.1093/gbe/evx078.
31. Michalovova, M.; Vyskot, B.; Kejnovsky, E. Analysis of plastid and mitochondrial DNA insertions in the nucleus (NUPTs and NUMTs) of six plant species: size, relative age and chromosomal localization. Heredity (Edinb) 2013, 111, 314-320, doi:10.1038/hdy.2013.51.
32. Bensasson, D.; Feldman, M.W.; Petrov, D.A. Rates of DNA duplication and mitochondrial DNA insertion in the human genome. J. Mol. Evol. 2003, 57, 343-354, doi:10.1007/s00239-003-2485-7.
33. Yan, Z.; Fang, Q.; Tian, Y.; Wang, F.; Chen, X.; Werren, J.H.; Ye, G. Mitochondrial DNA and their nuclear copies in the parasitic wasp Pteromalus puparum: A comparative analysis in Chalcidoidea. Int J Biol Macromol 2019, 121, 572-579, doi: 10.1016/j.ijbiomac.2018.10.039.
34. Wertheim, J.O.; Murrell, B.; Smith, M.D.; Kosakovsky Pond, S.L.; Scheffler, K. RELAX: detecting relaxed selection in a phylogenetic framework. Mol Biol Evol 2015, 32, 820-832, doi:10.1093/molbev/msu400.
35. Storchová, Z.; Breneman, A.; Cande, J.; Dunn, J.; Burbank, K.; O'Toole, E.; Pellman, D. Genome-wide genetic analysis of polyploidy in yeast. Nature 2006, 443, 541-547, doi:10.1038/nature05178.
36. Petrov, D.A.; Sangster, T.A.; Johnston, J.S.; Hartl, D.L.; Shaw, K.L. Evidence for DNA loss as a determinant of genome size. Science 2000, 287, 1060-1062, doi:10.1126/science.287.5455.1060.
37. Yoshida, T.; Furihata, H.Y.; Kawabe, A. Patterns of genomic integration of nuclear chloroplast DNA fragments in plant species. DNA Res 2014, 21, 127-140, doi:10.1093/dnares/dst045.
38. Goldin, E.; Stahl, S.; Cooney, A.M.; Kaneski, C.R.; Gupta, S.; Brady, R.O.; Ellis, J.R.; Schiffmann, R. Transfer of a mitochondrial DNA fragment to MCOLN1 causes an inherited case of mucolipidosis IV. Hum Mutat 2004, 24, 460-465, doi:10.1002/humu.20094.
39. Turner, C.; Killoran, C.; Thomas, N.S.; Rosenberg, M.; Chuzhanova, N.A.; Johnston, J.; Kemel, Y.; Cooper, D.N.; Biesecker, L.G. Human genetic disease caused by de novo mitochondrial-nuclear DNA transfer. Hum Genet 2003, 112, 303-309, doi:10.1007/s00439-002-0892-2.

| 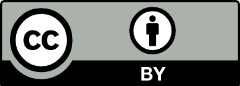 | © 2020 by the authors. Submitted for possible open access publication under the terms and conditions of the Creative Commons Attribution (CC BY) license (http://creativecommons.org/licenses/by/4.0/). |
| --- | --- |
